# A general framework for interpretable neural learning based on local information-theoretic goal functions

**DOI:** 10.1073/pnas.2408125122

**Published:** 2025-03-05

**Authors:** Abdullah Makkeh, Marcel Graetz, Andreas C. Schneider, David A. Ehrlich, Viola Priesemann, Michael Wibral

**Affiliations:** ^a^Department of Data-driven Analysis of Biological Networks, Göttingen Campus Institute for Dynamics of Biological Networks, University of Göttingen, Göttingen 37077, Germany; ^b^Complex Systems Theory, Max Planck Institute for Dynamics and Self-Organization, Göttingen 37077, Germany; ^c^Department of Chemistry and Applied Biosciences, ETH Zurich, Zurich 8092, Switzerland; ^d^University of Göttingen, Göttingen 37073, Germany; ^e^Cluster of Excellence “Multiscale Bioimaging: from Molecular Machines to Networks of Excitable Cells” (MBExC), University of Göttingen, Göttingen 37073, Germany

**Keywords:** information theory, partial information decomposition, neural networks, local learning

## Abstract

Despite the impressive performance of biological and artificial networks, an intuitive understanding of how their local learning dynamics contribute to network-level task solutions remains a challenge to this date. Efforts to bring learning to a more local scale indeed lead to valuable insights, however, a general constructive approach to describe local learning goals that is both interpretable and adaptable across diverse tasks is still missing. We have previously formulated a local information processing goal that is highly adaptable and interpretable for a model neuron with compartmental structure. Building on recent advances in Partial Information Decomposition (PID), we here derive a corresponding parametric local learning rule, which allows us to introduce “infomorphic” neural networks. We demonstrate the versatility of these networks to perform tasks from supervised, unsupervised, and memory learning. By leveraging the interpretable nature of the PID framework, infomorphic networks represent a valuable tool to advance our understanding of the intricate structure of local learning.

## Significance

Which learning goals must individual computational elements pursue to contribute to a network-level task solution? This local understanding is missing in both biological, but also artificial neural networks, despite their impressive performance. We address this question by characterizing the information processing motifs of individual neurons as local goal functions, derived from first principles of information theory. A simple parameterization then enables the definition of an abstract goal function that spans a broad space of different learning rules and tasks. The resulting “infomorphic” networks offer a constructive approach to understanding local learning and information processing in neural networks, creating a bridge between theoretical neuroscience and artificial intelligence.

Both biological neural networks (BNNs) and artificial neural networks (ANNs) are capable of solving a variety of complex tasks, thanks to their interconnected structure comprising a large number of similar computational elements. The human neocortex employs a variety of neuron types organized into canonical, repeating microcircuits that show high functional flexibility (1–3), similar to how ANNs utilize relatively simple processing units arranged in repetitive structures (4). This structural repetition combined with functional flexibility enables both types of networks to scale drastically in size and complexity. Given the high intrinsic complexity of these networks, achieving an interpretable understanding of how local computational elements coordinate to address global tasks is challenging and remains an ongoing focus of intense research for both BNNs (5, 6) and ANNs (7, 8). Despite advances toward mechanistic interpretability of the inner local computational structures that emerge through learning (9, 10), the insights gained from post hoc approaches are specific to the data and network architecture, limiting their generality.

To foster a more general understanding of the local structures in neural networks, a data-independent description of the local algorithm is favorable. Such a description can be achieved through identifying a local optimization goal or learning rule, which prioritizes the learning process over the resulting representation. Traditionally, local learning has largely been formulated from two general perspectives: On one hand, the experimental study of BNNs has revealed activity-dependent changes of synaptic strengths. This has led researchers to propose a remarkable variety of local learning rules (11–15), most of which focus on biologically plausible mechanisms and require only locally available information. Despite these efforts, building large and powerful networks using only these mechanistic local learning rules has proven challenging (16). On the other hand, local learning in ANNs typically emerges implicitly by setting network-wide goal functions to satisfy global task requirements and then optimizing the network parameters via nonlocal gradient optimization. Such an approach hinders insights at the local scale, as the description of neuron function remains purely arithmetic. Nonetheless, efforts have recently intensified in developing learning rules that are both local, i.e., relying only on information that is available at the site, and show potential for scaling to larger, more capable networks (16–18). This includes learning rules based on concepts from contrastive learning (19, 20), predictive coding (21–23), local information maximization (15, 24) and many others (25–30).

Despite this large variety of fruitful efforts toward more local forms of learning, most existing approaches are limited to specific learning paradigms and implementations. What seems to be missing is a unifying framework to describe local learning goals—general enough to be applied across learning paradigms, datasets, and implementations, while still being interpretable. A promising starting point for developing such a framework from first principles is information theory (31–36). From an information-theoretic perspective, the local computational elements in a neural network can be interpreted as information channels that convert incoming signals into outgoing activity (37), with the conversion being specified by their synaptic weights. Previous research has demonstrated the feasibility and potential of a framework of local information-theoretic goal functions based on a decomposition of the output information of the individual computational elements (32, 35).

However, since classical information theory is constructed from an information channel (simple input-to-output) perspective, it is fundamentally limited in its ability to describe all facets of information processing: Both the proposed biological learning in neurons and most proposed biologically plausible local learning in ANNs require at least two qualitatively different classes of inputs to the computational element, one carrying the information to be processed and the other one carrying contextual information on how to process it (e.g., feed-back, label, error, lateral, contrastive, or reward signals) (38–42). To be able to capture the general interactions that could arise between these two classes of inputs, Wibral et al. (31) proposed a generalization and unification of existing information-theoretic local goal functions by employing the more expressive and intuitive Partial Information Decomposition (PID). PID provides a comprehensive information-theoretic description of the complex interactions of multiple sources with respect to a target by dissecting the mutual information into unique, redundant, and synergistic contributions (43–46).^*^

**Fig. 1.**
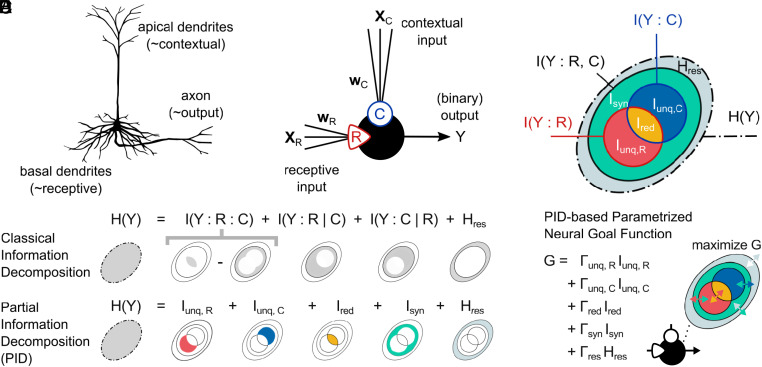
The infomorphic neuron model, analogous to cortical pyramidal neurons, separately integrates two distinct classes of inputs. The neuron adjusts its synaptic weights to maximize the local goal function G, based on an information-theoretic decomposition of its own output information. (*A*) Cortical pyramidal neurons with separate synaptic integration sites for basal and apical dendrites, the former driving output and the latter providing contextual modulation. (*B*) The infomorphic neuron, modeled after these neurons, is characterized by two functionally distinct sets of inputs that are scaled by synaptic weights and added to obtain the integrated input signals R (receptive) and C (contextual). R and C contribute individually to the probabilities of the neuron’s binary output, which are computed using an activation function A(R,C) and a sigmoid transformation function. (*C*) The total Shannon output information H(Y) of the neuron consists of the mutual information with the joint inputs I(Y:R,C) and additional residual information $H_{\mathrm{res}\;}=H\left(Y|R,C\right)$ that originates directly from the stochasticity of the neuron. Using Partial Information Decomposition (PID), the joint mutual information I(Y:R,C) can be further subdivided into four information contributions: i) $I_{\mathrm{red}\;}$, the redundant information that is provided by either R or C individually, ii) $I_{\mathrm{unq}\;,R}$, the unique information of R that is only provided by R but not by C, iii) $I_{\mathrm{unq}\;,C}$, the unique information of C that is only provided by C but not by R, and iv) $I_{\mathrm{syn}\;}$, the synergistic information that is provided by R and C only when taken jointly but neither by R nor C taken individually. (*D*) Any classical mutual-information-based decomposition can only provide a linear combination of the underlying PID quantities. (*D*, *Upper*) Classical decomposition into four information contributions that formed the basis for prior work (32–35): the coinformation I(Y:R:C), the two conditional mutual information values I(Y:R∣C) and I(Y:C∣R), and the stochasticity-caused residual entropy $H_{\mathrm{res}\;}$. (*D*, *Lower*) The five contributions that are quantified using PID. (*E*) The neuron’s synaptic weights w are optimized to maximize a goal function G that is based on the Partial Information Decomposition of the neuron’s overall output information H(Y) and parameterized by $\Gamma =(\Gamma_{\mathrm{unq}\;,R},\Gamma_{\mathrm{unq}\;,C},\Gamma_{\mathrm{red}\;},\Gamma_{\mathrm{syn}\;},\Gamma_{\mathrm{res}\;})$. Panel (*A*) is adapted from Fabian Mikulasch’s original depiction of a pyramidal neuron (21) (CC0).

For the case of two input classes, PID distinguishes four contributions to the overall information processing: Each class may contribute uniquely to the output, meaning they contribute information the other source does not have, they can provide redundant information or they could contribute synergistically, i.e., in a way that no input class can do alone. Taken by itself, each input class can only provide the redundant information and its unique contribution, while the synergy relies on access to both classes simultaneously (Fig. 1 *C* and *D*). Here, we argue that different learning paradigms require different processing of the local information from the two classes. For instance, in a supervised setting, where one class provides input data and the other provides the ground-truth labels, the intuitive goal becomes to encode in the output what is redundant between these two input classes, which enables the network to learn to extract the label information from the input signal. In general, the interpretability of these information atoms allows to intuitively identify which information processing is necessary at a local level to achieve a global task.

In this work, we derive a parametric local learning rule from a general PID-based goal function, leveraging the differentiable $i_{\cap }^{\mathrm{sx}\;}$ PID measure (45). We provide a proof of principle that this local learning rule enables networks consisting of compartmental neurons to solve tasks across three classic learning paradigms—supervised, unsupervised, and associative memory learning. Our work additionally shows that PID-based goals can be flexibly applied to different datasets and architectures, while being intuitively interpretable. Note that the relatively simple networks studied in this work should be considered an initial step toward larger and more capable network structures and provide evidence for the promising potential of such a general framework of interpretable local learning goals.

Below, we first explain our view of neurons as information processors with multiclass input, efficiently characterized by PID. Based on these insights, we then introduce a compartmental neuron model and apply it to a collection of learning scenarios. We conclude with a discussion of strengths, limitations, and next steps. As a side note, the neurons and networks developed in this work are termed infomorphic—as a portmanteau of “information” and “morphous” to indicate that they are directly shaped by the information they process.

## 1. Using Information Theory to Describe the Information Processing of a Neuron

In general, a neuron can be regarded as a Shannon information channel receiving synaptic inputs X from its afferent synapses and producing its own activity Y as output. Here, both X and Y are modeled as random variables and their relationship can be, in the general case, stochastic. The mutual information (52, 53)

$$\begin{array}{c} I\left(Y:X\right)={\;E}_{y,x}\log_{2}p\left(y|x\right)/p\left(y\right) \end{array}$$

then quantifies how much a neuron’s output is influenced by its synaptic inputs, whereas the residual (or conditional) entropy

$$\begin{array}{c} H\left(Y|X\right)=-{\;E}_{y,x}\log_{2}p\left(y|x\right) \end{array}$$

quantifies the amount of stochasticity in the output of the neuron that is not predictable from its inputs. The sum of these quantities equals the total entropy or information content of the firing of the neuron

[1] $$\begin{array}{c} H(Y)=I(Y:X)+H(Y|X). \end{array}$$

### 1.1. Beyond Simple Channels: Differentiating Input Classes.

The picture of neurons as simple information channels has to be refined in light of the insight that different information streams into a neuron often play qualitatively different roles. In ANNs, forward-propagated signal and backpropagated gradients influence the neuron in very different ways. Similarly, biological neurons often have multiple classes of inputs with distinct information processing characteristics (54). An example of a biological neuron with two distinct input classes can be found in layer-5 pyramidal neurons (55). These neurons are ubiquitous in the cortex, involved in sensory, cognitive, and motor tasks, and have been hypothesized to play a role in conscious awareness (2, 56). They are typically embedded in a relatively stereotyped cortical microcircuit, at the junction of feed-forward and feed-back information streams in the cortical hierarchy (57). To process these two information streams, pyramidal neurons possess two distinct types of dendrites, the basal and apical dendrites (55). Basal dendrites receive input from hierarchically lower cortical areas and play a role in encoding the external features of the environment that are processed along the cortical hierarchy (38). Apical dendrites, in contrast, receive contextual input from higher cortical areas and have been shown to play an important role in modulating perception (58, 59). This connectivity is similar across a range of different brain areas and cognitive domains, motivating the assumption that the general function of pyramidal neurons is independent of the semantics of their input (Fig. 1*A* and ref. 60).

The two-compartment structure of layer-5 pyramidal neurons (61) is consistent with many biologically plausible local learning rules in ANNs that require at least two qualitatively different classes of inputs to the neuron, respectively carrying feedforward information to be processed and contextual information to guide this processing (e.g., feed-back, label, error, lateral, contrastive, or reward signals) (38–42). Once these two input classes are explicitly established, they motivate a local learning goal.

To prepare for the mathematical representation of a neuron’s unique, redundant, and synergistic information, we will first reinterpret the source variable X from above as being a composite variable $X=(X_{R},X_{C})$ of the receptive input $X_{R}$, which is inspired by the input to the basal dendrites, and the contextual input $X_{C}$, which is inspired by the input to the apical dendrites. Analogous to Eq. **1**, the total entropy of the neuron Y can now be written as

[2] $$\begin{array}{c} H\left(Y\right)=I\left(Y:X_{R},X_{C}\right)+H\left(Y|X_{R},X_{C}\right). \end{array}$$

The dissection of X additionally allows to consider the individual channels of the receptive or contextual inputs to the target, which are characterized by the mutual information terms $I(Y:X_{R})$ or $I(Y:X_{C})$, respectively. Note, however, that these two channels do not simply add up to the total mutual information $I(Y:X_{R},X_{C})$, because the sum $I\left(Y:X_{R}\right)+I\left(Y:X_{C}\right)$ contains information which is redundantly present in both input classes and will be double-counted, while synergistic information which only becomes apparent if one considers $X_{C}$ and $X_{R}$ simultaneously will be overlooked (see Fig. 1*C*, 53, 62). By introducing the coinformation

[3] $$\begin{array}{cc} I(Y:X_{R}:X_{C}) & =I(Y:X_{R},X_{C})\\ & \;-I\left(Y:X_{R}|X_{C}\right)-I\left(Y:X_{C}|X_{R}\right), \end{array}$$

the decomposition in Eq. **2** can be refined to

[4] $$\begin{array}{cc} H(Y) & =I(Y:X_{R}:X_{C})\\ & \;+I\left(Y:X_{R}|X_{C}\right)+I\left(Y:X_{C}|X_{R}\right)\\ & \;+H(Y|X_{R},X_{C}), \end{array}$$

where the conditional mutual information $I(Y:X_{R}|X_{C})$ captures the remaining dependence of Y on $X_{R}$ when $X_{C}$ is known, and $I(Y:X_{C}|X_{R})$ is defined analogously (53).

Kay used this decomposition as the starting point to construct models of learning neurons with information theoretic objective functions (32). In our work, we build on this concept by exploiting the superior expressiveness provided by the framework of Partial Information Decomposition to build infomorphic neurons.

### 1.2. Uncovering the Information Processing Between Different Input Classes Using Partial Information Decomposition.

The above perspective of viewing a neuron as a collection of information channels still paints an incomplete picture of the information processing within a neuron because it cannot account for all the different ways in which the different information sources combine and determine the output information: While some of the information in the neuron’s output activity Y might be provided uniquely by either the receptive input $X_{R}$ or the contextual input $X_{C}$, other parts might be redundantly supplied by both of them while yet others only become available synergistically when both sources are considered jointly (31). Classical information theory is insufficient for this distinction as it has no concept of “sameness” of information: While one can compute the total amount of information in the output that is coming from each source or from both sources together using mutual information, there is no way of quantifying how much of the information contributed to the output is the same, i.e., redundantly provided by the input variables about the output (43).

Dissecting the mutual information between multiple source variables and a single target variable into nonoverlapping additive information atoms is the subject of Partial Information Decomposition (43, 46). Using PID, we can subdivide the entropy H(Y) into five parts (Fig. 1*C*)

[5] $$\begin{array}{cc} H(Y) & =I_{\mathrm{unq}\;}\left(Y:X_{R}\right)+I_{\mathrm{unq}\;}\left(Y:X_{C}\right)\\ & \;+I_{\mathrm{red}\;}\left(Y:X_{R},X_{C}\right)+I_{\mathrm{syn}\;}\left(Y:X_{R},X_{C}\right)\\ & \;+H(Y|X_{R},X_{C}), \end{array}$$

where $I_{\mathrm{unq}\;}\left(Y:X_{R}\right)$ and $I_{\mathrm{unq}\;}\left(Y:X_{C}\right)$ are the unique information atoms of the receptive and contextual inputs, respectively, $I_{\mathrm{red}\;}\left(Y:X_{R},X_{C}\right)$ refers to the redundant (shared) information, and $I_{\mathrm{syn}\;}\left(Y:X_{R},X_{C}\right)$ refers to the synergistic (complementary) information. These four atoms can describe the information processing in Y of $X_{R}$ and $X_{C}$ in versatile ways, while also having meaningful interpretations: For example, if a neuron encodes the coherent parts of its inputs, this would be reflected in a high redundant information. Alternatively, a neuron might encode the information in its receptive input $X_{R}$ that is specifically not present in the contextual input $X_{C}$, which would translate to a high unique information contribution from $X_{R}$. Finally, if the neuron’s output contains information which cannot be obtained from any single source alone, for instance if the output Y reflected the logical “exclusive or” of its inputs, the synergy between the sources would be high. Overall, PID provides a decomposition framework with well-defined and intuitive interpretations for understanding a neuron’s information processing.

Note that while the coinformation $I(Y:X_{R}:X_{C})$ (in Eq. **3**) is equal to the difference between redundant and synergistic information

[6] $$\begin{array}{c} I\left(Y:X_{R}:X_{C}\right)=I_{\mathrm{red}\;}\left(Y:X_{R},X_{C}\right)-I_{\mathrm{syn}\;}\left(Y:X_{R},X_{C}\right), \end{array}$$

classical information theory provides no tool to disentangle the two components.

To analyze the information processing of a neuron, the aforementioned PID atoms need to be quantified. Note that despite their strong relation to classical information-theoretic quantities through Eqs. **5** and **6**, the size of the PID atoms cannot be determined from classical information-theoretic quantities alone as there are four atoms with only three equations providing constraints (43). An additional quantity has to be defined for PID, which is typically, but not necessarily, the redundant information (43, 44, 46, and references therein). By now, a multitude of different measures for redundant information have been proposed, each fulfilling a number of partly mutually exclusive desiderata and drawing on concepts from different fields such as decision or game theory (44, 63, 64, 65, 66). In this work, we use the PID measure $I_{\cap }^{\mathrm{sx}\;}$ defined by Makkeh et al. (45) due to its analytical differentiability with respect to the underlying joint probability distribution $\;P(Y,X_{R},X_{C})$, allowing for optimization of the PID quantities through gradient ascent.

## 2. Infomorphic Neurons

In a line of similar work, Kay (32) utilized the decomposition in Eq. **4** not as a post hoc analysis tool, but as a parameterizable optimization goal function, extending this idea in subsequent research (33–35). Even before the development of their differentiable PID measure, Wibral et al. (31) envisioned a similar, but more refined neural goal function derived from the decomposition in Eq. **5**. In the following paragraphs, we realize this idea in a neuron model closely aligned to prior work (32), which we refer to as the infomorphic neuron, and derive analytic gradients for the PID-based goal function.

### 2.1. Multicompartment Computation.

Infomorphic neurons operate in discrete time and output values Y∈{−1,+1} (referred to as “LOW” and “HIGH”), in analogy to time-binned spike trains of biological neurons. Akin to the basal and apical dendrites of layer-5 pyramidal neurons, an infomorphic neuron distinguishes between two classes of input synapses, namely “receptive” inputs $X_{R}$ and “contextual” inputs $X_{C}$ (Fig. 1 *A* and *B*). Inspired by how the inputs of different input classes are individually aggregated in separate compartments in these biological neurons (55), the inputs of the two classes of the infomorphic neuron are separately combined in a weighted sum to produce the aggregate inputs $R=w_{R}^{T}X_{R}-w_{0,R}$ and $C=w_{C}^{T}X_{C}-w_{0,C}$ (32). Here, $w_{R}$ and $w_{C}$ reflect the weights associated with the receptive and contextual inputs, respectively, while $w_{0,R}$ and $w_{0,C}$ denote constant bias values. At any time step, the probability θ of a neuron to be in the HIGH state depends only on the instantiation of its aggregate inputs r and c, as follows:

$$\begin{array}{c} \theta (r,c):=\;P(Y=1|R=r,C=c):=\sigma (A(r,c)), \end{array}$$

where $\sigma \left(\xi \right)=1/\left(1+e^{-\xi }\right)$ is a sigmoid nonlinearity, and A is an additional activation function. While the activation function can in principle be chosen arbitrarily, a biology-inspired choice of A may draw inspiration from layer-5 pyramidal neurons: By making the activation function be primarily dependent on the receptive inputs, one can imitate the privileged role that basal dendrites play in driving pyramidal neurons (33). In practice, we adapted the degree to which the contextual input influences the output, dependent on the requirements of each task. The choice of activation function will be individually motivated and discussed in the corresponding experimental sections.

### 2.2. Local Learning.

Each infomorphic neuron optimizes its local information processing by changing the two sets of weights $w_{R}$ and $w_{C}$ of its incoming (afferent) synapses. This information processing can take on very different shapes: For some tasks, optimal information processing could mean coding for coherence between the receptive and contextual inputs, while for other tasks, optimal processing might entail extracting any piece of information (e.g., a feature) exclusively provided by the receptive inputs that is not present in the contextual input.

Kay first derived a local goal function from an information-theoretic partition of the local mutual information of a neuron (32). Here, we argue for a similar local goal function involving a linear combination of the five components of the output entropy of a neuron as derived from PID and first established by (31):

[7] $$\begin{array}{cc} G(Y:\tilde{R},\tilde{C}) & =\Gamma_{\mathrm{unq}\;,R}\;I_{\mathrm{unq}\;}\left(Y:\tilde{R}\right)+\Gamma_{\mathrm{unq}\;,C}\;I_{\mathrm{unq}\;}\left(Y:\tilde{C}\right)\\ & \;+\Gamma_{\mathrm{red}\;}\;I_{\mathrm{red}\;}\left(Y:\tilde{R},\tilde{C}\right)+\Gamma_{\mathrm{syn}\;}\;I_{\mathrm{syn}\;}\left(Y:\tilde{R},\tilde{C}\right)\\ & \;+\Gamma_{\mathrm{res}\;}\;H\left(Y|\tilde{R},\tilde{C}\right)\\ & =:(\Gamma_{\mathrm{unq}\;,R},\Gamma_{\mathrm{unq}\;,C},\Gamma_{\mathrm{red}\;},\Gamma_{\mathrm{syn}\;},\Gamma_{\mathrm{res}\;})\\ & \;\cdot {\left(I_{\mathrm{unq}\;,R},I_{\mathrm{unq}\;,C},I_{\mathrm{red}\;},I_{\mathrm{syn}\;},H_{\mathrm{res}\;}\right)}^{T}. \end{array}$$

Here, the variables $\tilde{R}$ and $\tilde{C}$ are binned versions of the continuous-valued R and C inputs, necessary due to the lack of a differentiable PID measure for mixed discrete-continuous variables (67) and other conceptual difficulties of information theory in continuous networks (68, 69). Note that the binning procedure itself, while used in analogy to previous work (32, 35), is a nondifferentiable operation whose gradients we do not take into account here. Future work might circumvent this problem by using parametric (e.g., bivariate Gaussian) approximations of p(R,C) (32, 35) combined with PID-estimators for mixed discrete-continuous variables. The neuron’s local goal function G is a linear combination of the PID atoms that is defined a priori by choosing the parameters Γ (Fig. 1*E*). In the second equality, we introduced a short-hand vector notation of G.

### 2.3. Optimizing the Goal Function.

The differentiability of the $I_{\cap }^{\mathrm{sx}}$ measure allows each neuron in an infomorphic network to optimize its own goal function G through gradient ascent.

The empirical gradients of G with respect to the weight vectors $w_{R}$ and $w_{C}$ can be analytically derived as

[8] $$\begin{array}{c} \frac{\partial \hat{G}}{\partial w_{R}}=\frac{1}{N}\sum_{x_{R},x_{C}}f_{p(R,C)}^{\Gamma }\left(\tilde{r},\tilde{c}\right)\;{\left.\frac{\partial A}{\partial r}\right|}_{\tilde{r},\tilde{c}}\;x_{R} \end{array}$$

and

[9] $$\begin{array}{c} \frac{\partial \hat{G}}{\partial w_{C}}=\frac{1}{N}\sum_{x_{R},x_{C}}f_{p(R,C)}^{\Gamma }\left(\tilde{r},\tilde{c}\right)\;{\left.\frac{\partial A}{\partial c}\right|}_{\tilde{r},\tilde{c}}\;x_{C}, \end{array}$$

where $\hat{G}$ indicates the estimator of G based on N input samples in the data, and f is implicitly dependent on the full probability distribution $p_{\tilde{R},\tilde{C}}$ of $\tilde{r}$ and $\tilde{c}$ over the dataset and the explicit current values of those variables, as well as the goal parameter vector Γ. The full derivation of the gradients can be found in *SI Appendix*, section 1.

In practice, we only update the network parameters after a fixed number of discrete network time steps, referred to as a minibatch. For each minibatch, we estimate the full binned probability distribution $p_{\tilde{R},\tilde{C}}$ from the histogram of inputs and finally conduct a single weight update. We report the number of minibatches as the training time t. Instead of using minibatches, it would also be possible due to the pointwise nature of the $i_{\cap }^{\mathrm{sx}\;}$ PID measure to keep running estimates with exponential forgetting of past samples, which would allow for weight updates after each network time step.

## 3. Infomorphic Networks Encompass Various Learning Paradigms

The parameterized information-theoretic goal function enables groups of infomorphic neurons, i.e., “infomorphic networks,” to serve as a very general and versatile approach to learning. In the following, we demonstrate their broad applicability by providing three example applications of infomorphic networks, on supervised learning, unsupervised learning, and online learning of associative memories.

In correspondence to classical ANNs, infomorphic networks require choices on network topology, activation functions, and goal functions, where the latter are chosen by setting the goal function hyperparameters for each neuron. The ability to arbitrate between different local goals by setting these hyperparameters is a major strength of our framework, and we will motivate and discuss our specific hyperparameter choices in all three presented applications.

### 3.1. Supervised Learning by Encoding Coherence Between Input and Label Information.

We construct a single-layer infomorphic network for supervised classification of MNIST digits (70).

#### 3.1.1. Topology and inputs.

**Fig. 2.**
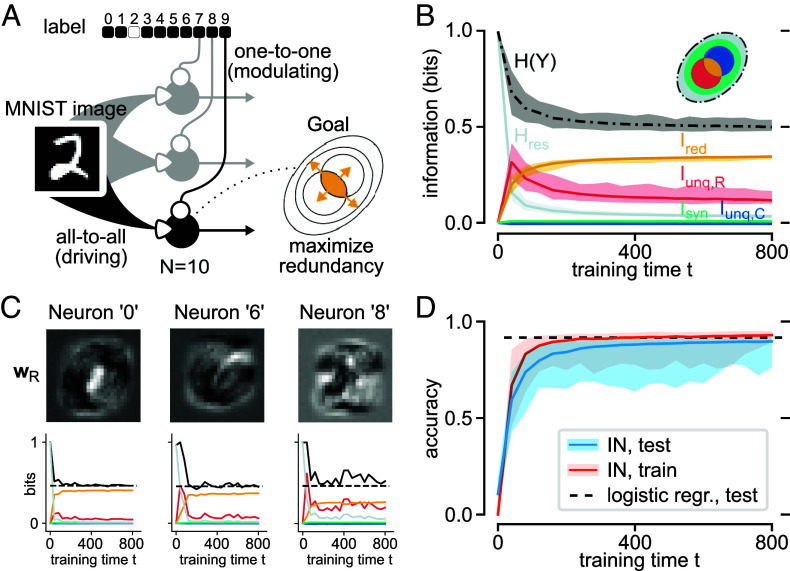
Supervised learning in single-layer infomorphic networks. By maximizing redundant information between image and label, the neurons learn to identify MNIST digits with a test accuracy comparable to logistic regression. (*A*) Network architecture with one-hot encoded label and 10 neurons, each receiving all 28 × 28 image pixels as $X_{R}$ and one element of the label vector as $X_{C}$. Activation function A(r,c):=r(0.5+σ(2rc)) is chosen such that c has only modulating effect on the binary output probabilities, in line with the label’s role as context for learning. The goal of each neuron is to transmit maximal information $I_{\mathrm{red}\;}$ that is redundant between R (image) and C (label element), thereby learning to act as a detector of its respective digit. The learning shows best stability if the goal function sets weak incentives for additionally maximizing the unique and synergistic information: $\Gamma_{\mathrm{supervised}}=\left(0.1,0.1,1,0.1,0\right)$. (*B*) Information quantities averaged over all neurons, shown for 100 independent training runs. (*C*) Receptive fields ($w_{R}$) and information quantities for three sample neurons for a single training run, the dotted line indicating the expected H(Y) in case of perfect classification (one-vs.-all entropy of label in test dataset). (*D*) The average training and test accuracies across 100 training runs, with test accuracy approaching that of logistic regression (reaching on average 89.7% vs. 91.9% for log. regr.). Note that in (*B*) and (*D*) the 95-percentile is being displayed.

Each of k=10 neurons receives the full MNIST image via a set of 28·28=784 receptive input synapses, with $X_{R}\in {\left\{0,\frac{1}{255},\cdots ,1\right\}}^{28\times 28}$, and a single element of a one-hot label vector as contextual input, with $X_{C}\in \left\{-1,1\right\}$ (Fig. 2*A*). In this setup, each neuron becomes a one-vs.-all classifier of its assigned digit.

#### 3.1.2. Goal functions.

Viewed through the lens of PID, supervised learning requires extracting from input data the same information that is contained in the label. This is achieved if each neuron’s output information is redundantly determined by its two input classes, motivating the goal function $G=I_{\mathrm{red}\;}$. In practice, weak incentives for the other PID quantities $\Gamma_{\mathrm{supervised}}=\left(\Gamma_{\mathrm{unq}\;,R},\Gamma_{\mathrm{unq}\;,C},\Gamma_{\mathrm{red}\;},\Gamma_{\mathrm{syn}\;},\Gamma_{\mathrm{res}\;}\right)=\left(0.1,0.1,1,0.1,0\right)$ improve performance and stability of learning by preventing neurons from going silent, i.e., always outputting the same value.

#### 3.1.3. Activation functions.

To ensure that the receptive inputs are strong enough to drive the neurons during the test phase when the label is missing (all label input $x_{C}=0$), we set the activation function to $A_{\sigma }\left(r,c\right):=r\left(0.5+\sigma \left(2rc\right)\right)$. This makes the binary output probabilities mostly dependent on the receptive input and only weakly modulated by the contextual information, rendering the label input a teacher signal that strongly influences learning but hardly the dynamics.

#### 3.1.4. Protocol.

In the training phase, we present the MNIST images and labels sequentially in random order, with a weight update after each minibatch (See *SI Appendix*, section 2.B for all chosen training parameters). In the test phase, we present previously unseen MNIST images and set the contextual input to $x_{C}=0$, calculating winner-take-all classification accuracy. Note that instead of calculating P(Y∣r,c=0), an optimal predictor would marginalize over $\;P(c_{\mathrm{train}})$ to estimate $\;P\left(Y|r\right)\approx \sum_{c_{\mathrm{train}}}\;P\left(Y|r,c_{\mathrm{train}}\right)\;P\left(c_{\mathrm{train}}\right)$. However, this would require running each test input twice for the two different labels, and performing computations that infomorphic neurons do not implement. Fortunately, due to the merely modulating role of the contextual input, the test accuracy of both approaches is virtually identical (see *SI Appendix*, section 3.A and Fig. S3).

#### 3.1.5. Performance and outcome.

The infomorphic networks reach an average test accuracy of 89.7% (Fig. 2*D*), slightly lower than the 91.9% we find for multinomial logistic regression. Indeed, logistic regression upper-bounds the network performance, because by setting $x_{C}=0$ in the test phase, the activation function simplifies to A(r,c=0)=r and the firing probability becomes $\theta =\sigma (w_{R}\cdot x_{R}-w_{0,R})$, identical to logistic regression. The receptive weights $w_{R}$ of individual neurons after training, plotted as receptive fields, visually reveal their assigned digit and qualitatively match the corresponding receptive fields found in logistic regression (Fig. 2*C* and *SI Appendix*, section 3.A and Fig. S2).

#### 3.1.6. Information dynamics.

Analyzing the information atoms of individual neurons over the course of training, we find an expected increase in redundant information $I_{\mathrm{red}\;}$ (Fig. 2 *B* and *C*). This increase is less pronounced in neurons corresponding to digits that are more likely to be confused (*SI Appendix*, section 3.A and Fig. S1). For these neurons we also find higher unique information from the receptive input $I_{\mathrm{unq}\;,R}$, indicating that they are still encoding image information that is not present in their label. Additionally, the average output entropy H(Y) of the neurons decreases and approaches the average entropy of the one-hot label encoding (label k present vs. absent) of 0.47 bits, while the residual entropy H(Y|R,C) decreases fast, reflecting a decrease in the neurons’ stochasticity.

### 3.2. Unsupervised Learning of Independent Features by Maximizing Each Neuron’s Unique Information About the Stimulus.

We construct a very simple data compression task that requires recurrent communication between neurons.

#### 3.2.1. Topology and inputs.

**Fig. 3.**
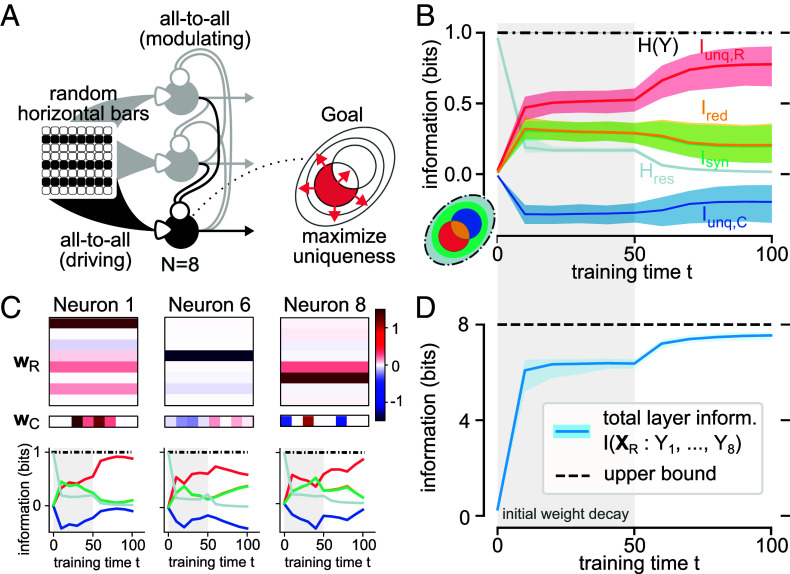
Unsupervised feature learning in recurrent infomorphic networks. By maximizing unique information with respect to all other neurons, the neurons self-organize to create a highly informative representation of the input. (*A*) Network architecture for unsupervised learning with 8 neurons, each receiving 8 × 8 binary pixel inputs as $X_{R}$ and output of all other neurons as $X_{C}$. The image consists of 8 independent horizontal bars, randomly appearing with P=0.5. Activation function A(r,c):=r(0.5+σ(2rc)) is chosen such that c has only modulating effect on the binary output probabilities, which leads to recurrent connections mainly acting as context for learning. The goal of each neuron is set to maximize unique information $I_{\mathrm{unq}\;,R}$ of its own receptive input R with respect to the output of all other neurons, received as contextual input C: $\Gamma_{\mathrm{unsupervised}}=\left(1,0,0,0,0\right)$. (*B*) Information quantities averaged over all neurons for 300 independent training runs, showing two-phase training for feature competition and stabilization. (*C*) Receptive and contextual fields ($w_{R}$, $w_{C}$) and information quantities of three sample neurons for a single training run. (*D*) Mutual information $I(X_{R}:Y_{1},...,Y_{8})$ between all neurons’ outputs and input image, approaching full encoding capacity and input information content of 8 bits. Note that in (*B*) and (*D*) the 95-percentile is being displayed.

Each of the k=8 neurons receives 8×8-pixel binary images as receptive inputs, $X_{R}\in {\left\{-1,1\right\}}^{8\times 8}$, with each image containing 8 horizontal bars appearing independently with probability P=0.5 (Fig. 3*A*). As contextual input, each neuron receives the activity of all other neurons in the previous time step, with $X_{C}\in {\left\{-1,1\right\}}^{7}$.

#### 3.2.2. Goal functions.

The network-level goal is to encode all 8 bits of the information provided by the image distribution, distributed over the neurons. This can be achieved if each neuron encodes one full bit of image information that is not already encoded by the other neurons, and motivates a goal function maximizing for the conditional mutual information $G=I\left(Y:R|C\right)=I_{\mathrm{unq}\;,R}+I_{\;\mathrm{syn}\;}$. In order to encourage the network to explicitly disentangle the contributions of each neuron, we chose to only encourage the unique information of the receptive input: $\Gamma_{\mathrm{unsupervised}}=\left(\Gamma_{\mathrm{unq}\;,R},\Gamma_{\mathrm{unq}\;,C},\Gamma_{\mathrm{red}\;},\Gamma_{\mathrm{syn}\;},\Gamma_{\mathrm{res}\;}\right)=\left(1,0,0,0,0\right)$.

#### 3.2.3. Activation functions.

To avoid temporal oscillations in the network, we chose the same activation function $A_{\sigma }\left(r,c\right):=r\left(0.5+\sigma \left(2rc\right)\right)$ as in the supervised context, making the recurrent connections relevant for learning, but less so for the dynamics.

#### 3.2.4. Protocol.

We sequentially present randomly sampled images containing between 0 and 8 bars. Due to the time delay in recurrent connections, presentation of a new image introduces a mismatch between the receptive and contextual inputs of all neurons. We compensate for the resulting noise by presenting each image for 8 successive time steps.

Early in training, neurons compete for which information to encode. Two neurons choosing to encode for the same bar leads to a local optimum where both neurons try to increase their receptive weights to reduce stochasticity, however cannot obtain high unique information. To avoid this local maximum and prolong the critical initial phase of high stochasticity and competition, we introduce a strong weight decay (linear down-scaling of all receptive weights after each time step) during the first half of training (see *SI Appendix*, section 2.C for all chosen training parameters).

#### 3.2.5. Performance and outcome.

Over the course of training almost all neurons learn to encode mutually different individual bars (Fig. 3*C*). Rare encoding errors occur exclusively when two neurons encode the same bar (*SI Appendix*, Fig. S8). Correspondingly, the total mutual information of the layer $I(X_{R}:Y_{1},\ldots ,Y_{8})$ approaches the entropy of the dataset, indicating successful compression of the receptive input information (Fig. 3*D*).

#### 3.2.6. Information dynamics.

The average unique receptive information of the neurons $I_{\mathrm{unq}\;,R}$ converges to 0.77 bits, with the average conditional mutual information $I\left(Y:R|C\right)=I_{\mathrm{unq}\;,R}+I_{\mathrm{syn}\;}$ reaching close to 1.0 bits (Fig. 3 *B* and *C*). This suboptimal result is mostly attributable to individual neurons not having fully converged onto their chosen bar and to the weak contextual cross-talk between neurons (*SI Appendix*, section 3.B and Fig. S4). However, the above-mentioned rare encoding errors, i.e., two neurons encoding the same bar, show a strikingly different signature of low unique information $I_{\mathrm{unq}\;,R}$ and high redundancy $I_{\mathrm{red}\;}$ (*SI Appendix*, section 3.B and Fig. S7).

### 3.3. Online Associative Memory Learning by Maximizing the Local Coherence Between Network Firing and External Input.

We construct an (auto)associative memory network, similar to the Hopfield network (71), with an infomorphic online learning rule.

#### 3.3.1. Topology and inputs.

**Fig. 4.**
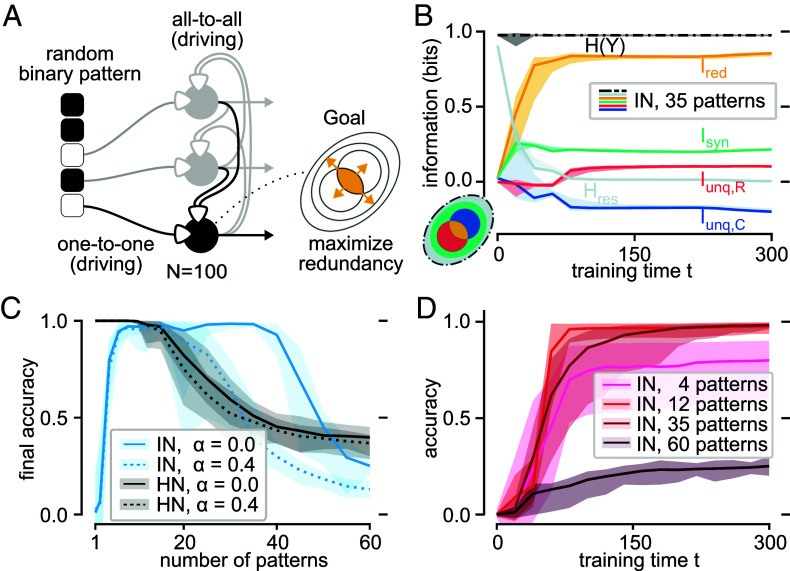
Associative memory learning in recurrent infomorphic networks. By maximizing redundant information between external input and output of all other neurons, the neurons learn to memorize a maximum number of patterns exceeding that of classical Hopfield networks. (*A*) Network architecture for memorizing binary patterns with 100 recurrently connected neurons, each receiving single element of target patterns as $X_{R}$ and output of all other neurons as $X_{C}$. Activation function A(r,c) is chosen such that both r and c can directly drive the neuron’s output probabilities, to enable an active recovery of patterns based only on recurrent connections at test time (Section 3.3). The goal of each neuron is to learn to predict their respective element of the pattern based on activity of other neurons, which can be done by maximizing the redundant information in the output. Similar to the supervised example, the goal function used here includes additional, weak incentives for maximizing unique and synergistic PID contributions: $\Gamma_{\mathrm{memory}}=\left(0.1,0.1,1,0.1,0\right)$. (*B*) Information quantities averaged over all neurons, for 25 independent runs trained on 35 memory patterns. (*C*) Final accuracy of infomorphic networks and classical Hopfield networks over different numbers of patterns on the horizontal axis. Shown are the results for two noise levels used for testing the recovery of memorized patterns. (*D*) Accuracy over training for all 25 runs for different numbers of patterns. Note that in (*B*–*D*) the 95-percentile is being displayed.

Each of k=100 neurons receives a single element of a (P=0.5)-sparse memory vector as receptive input, with $X_{R}\in \left\{-1,1\right\}$, and the activity of all other neurons in the previous time step as contextual input, with $X_{C}\in {\left\{-1,1\right\}}^{99}$ (Fig. 4*A*).

#### 3.3.2. Goal functions.

The network-level goal is to align with any external input, when present, and over time inscribe it as an attractor, i.e., a memory, into the recurrent dynamics. The external input thus functions as both the memory cue and the teaching signal, depending on the duration of presentation. Learning a memory pattern upon repeated presentation implies that the recurrent contextual inputs learn to align with the external receptive inputs by providing the same information about the firing of a neuron as the external inputs, motivating the goal function $G=I_{\mathrm{red}\;}$. As in the supervised experiment, weak incentives for the other PID quantities $\Gamma_{\mathrm{memory}}=\left(\Gamma_{\mathrm{unq}\;,R},\Gamma_{\mathrm{unq}\;,C},\Gamma_{\mathrm{red}\;},\Gamma_{\mathrm{syn}\;},\Gamma_{\mathrm{res}\;}\right)=\left(0.1,0.1,1,0.1,0\right)$ improve performance by preventing neurons from going silent.

#### 3.3.3. Activation functions.

In the absence of a receptive input the neurons should be driven by the contextual synapses, while receptive input, if present, should overrule this recurrent drive and force the neurons into a new firing pattern. As a consequence, each input class individually needs to be able to drive the neuron. To make both inputs driving and encourage high weights, we choose the symmetric activation function

$$\begin{array}{c} A\left(r,c\right)=\frac{r^{8}\mathrm{sign}\left(r\right)+c^{8}\mathrm{sign}\left(c\right)}{r^{8}+c^{8}}\cdot {\left(r^{8}+c^{8}\right)}^{1/8} \end{array}$$

that is monotonic in both r and c and aligns with the positive and negative 8-norm for r,c>0 and r,c<0, respectively. (See *SI Appendix*, section 2.D for all chosen training parameters).

#### 3.3.4. Protocol.

In the training phase, we sequentially presented a set of memory patterns to the network in random order. As in the unsupervised experiments, to compensate for the time delay of the recurrent connections, we presented each memory pattern for 8 consecutive time steps. Note that structured sequences of patterns presented for only one time step lead to heteroassociative memory formation (results not reported here, but see ref. 72).

In the test phase, we present noise-corrupted memory patterns for a single time step, with the noise level 0≤α≤1 indicating the fraction of pattern elements set at random. After presentation, we set the receptive input of all neurons to $x_{R}=0$ and assess retrieval accuracy by computing the cosine similarity between the network state and the noiseless memory pattern after 20 time steps (Fig. 4*D*). We define the noise-dependent capacity of the network as the highest number of trained memory patterns such that the average retrieval accuracy exceeds 95%.

#### 3.3.5. Performance and outcome.

For noiseless initialization, infomorphic networks attain a capacity of 35 patterns per 100 neurons, which significantly exceeds the limit of 14 patterns in classical Hopfield networks (71). Note that unlike Hopfield networks our readout includes no binarization but remains stochastic, thus likely even limiting the measured capacity in the direct comparison. Furthermore, infomorphic networks outperform Hopfield networks up to a noise level of α=0.4, making them far more robust to random pattern distortion, even though training is conducted on noise-free patterns (Fig. 4*C*). Interestingly, infomorphic networks cannot reliably encode very few patterns, as in this case, many neurons receive the exact same input for every pattern, resulting in 0 bits of information in the receptive inputs.

#### 3.3.6. Information dynamics.

We find an expected increase in redundant information $I_{\mathrm{red}\;}$ over the course of training (Fig. 4*B*). Surprisingly, this increase is also present in networks that are seemingly above capacity, but then coincides with misinformative unique contextual information $I_{\mathrm{unq}\;,C}<0$, indicating that each neuron’s activity is not fully predictable by the other neurons in this case (*SI Appendix*, section 3.C and Fig. S9).

## 4. Discussion

In this work, we defined the infomorphic neuron, an artificial neuron with two input classes and a flexible, parameterized local goal function derived from Partial Information Decomposition (43). Like classical information theory, PID provides an abstract, high-level description of neural functioning, yet enriches this description with the additional structure of redundancy, uniqueness, and synergy. This structure is inherited by the infomorphic neuron and leads to a highly interpretable and flexible description of local goals, independent of task, substrate, type of signals, and encoding of information therein (31, 37).

The experiments conducted provide a proof of principle that the level of abstraction gained by an information-based approach does not compromise the ability of model neural networks to learn and solve diverse tasks. Concretely, we find that maximizing the encoding of redundant information between the input and the label enables a single-layer network of infomorphic neurons to do supervised learning. Furthermore, we show that input information can be distributed between multiple infomorphic neurons in a recurrent network in an unsupervised learning task, by making each neuron maximize its encoding of unique input information with respect to the activity of other neurons. Finally, we find that maximizing the encoding of redundant information between an external input and the activity of other neurons in a recurrent infomorphic network leads to the formation of robust associative memories that exceed the memory capacity of classical Hopfield networks.

Notably, these experiments only explore a fraction of the available space of parameterized goal functions, and other terms of the goal function might become relevant in other learning scenarios. To demonstrate this, we construct primitive error neurons in the spirit of predictive coding (23, 73) (*SI Appendix*, section 4.B and Fig. S10), which receive simulated observations as receptive input and simulated model predictions as contextual input. They are trained to maximize synergistic information and both unique information atoms, while simultaneously minimizing redundant information. In a similar spirit, goals can be linearly combined to create more complex goal functions in other scenarios. One example of this is the weak incentives for unique and synergistic atoms in the supervised and associative memory experiments. We expect the residual entropy $H_{\mathrm{res}\;}$ to be another useful incentive to either reduce or artificially inject noise into infomorphic networks to improve learning.

Note that changing the hyperparameters Γ and the activation functions allows us to arbitrate between three very different learning tasks with little effort, providing practical evidence that our goal function G is highly interpretable and provides an intuitively accessible understanding of the local goal of each neuron in solving various tasks. Such interpretability is hard to establish in conventional ANNs, where a global error minimization goal is automatically backpropagated to the local level to adjust neuron parameters (e.g., refs. 74 and 75). Furthermore, a similar understanding of local goal functions might be an insightful target in our description of biological neural networks, and ultimately help to bridge the gap between artificial and biological intelligent systems. To this end, the synthetic methodology of infomorphic networks can easily be combined with post hoc analyses of trained BNNs and ANNs. In particular, it remains an open question which local information quantities these existing networks are effectively maximizing (37, 47, 49).

### 4.1. Future Work.

Both the supervised and unsupervised experiments reported here focused on small single-layer neural networks, yet the ultimate strength of neural networks lies in their scalability to multilayered networks with a large number of neurons (4). Currently, this scalability is not present in infomorphic networks due to a conundrum that is implicitly solved in backpropagation: In most architectures, a neuron does not only need to get a feedforward input and a context signal that conveys target information (like a reward, supervision, or self-supervision signal), but additionally it requires knowledge about what other neurons in the same layer are coding for, such that it can choose to provide a complementary contribution with respect to the target (22). In backpropagation, this information flows to the neuron implicitly through the gradient signal from higher layers (22, 76), yet in infomorphic networks, it needs to be provided explicitly, because it fulfills a different role from the feed-back information: While the neuron typically needs to follow the feed-back signal (redundancy), it simultaneously needs to be different from the lateral signal (uniqueness). In follow-up work, we define an infomorphic neuron with three input classes that combines these ideas, leading to greatly improved supervised learning performance (77).

Additionally, it has been shown that encoding for synergy plays a role in integration of information from multiple sources or sensory streams, both in the brain (78, 79) and in ANNs (80). This could be tested constructively in infomorphic neurons with four input classes, which could be trained to extract information that is synergistic between two different receptive inputs, redundant with a contextual input and unique with respect to other lateral neurons.

Infomorphic networks also offer a natural connection to other information-based learning algorithms. Prominently, under the infomax principle (81) it has been shown that in the low-noise regime, neurons maximize global information encoding by finding unique, independent features, which is in line with uniqueness maximization in our unsupervised learning experiments. However, under high noise, as might be prevalent in biological networks (82), more cooperative, redundant representations emerge (81). It remains an intriguing open question whether increasing admixtures of redundancy to the individual neural goals of infomorphic neurons may lead them to develop similar noise-robust representations. To test this hypothesis, our current framework allows increasing the noise by introducing an additional $H_{\mathrm{res}\;}$ term or stronger weight decay. However, learning under constant noise is a constrained optimization problem and we leave the introduction of the required Lagrange multipliers to future work.

Despite their fully local computation, infomorphic neurons currently lack biological plausibility due to the complexity of the gradient equations (Eqs. **8** and **9**) as well as the memory-expensive histogram estimation method in Section 2. Whether the gradients can be effectively approximated by simpler equations remains an open question for future work. However, a parameterized estimation of p(R,C) combined with PID-estimators for mixed discrete-continuous variables would significantly reduce memory cost, e.g., to only 5 parameters for a two-dimensional Gaussian (32, 35), while for some tasks like supervised classification, more expressive multimodal distributions might be necessary. In addition, infomorphic neurons are functional, not anatomical, units and might thus be modeled by small microcircuits instead of individual spiking neurons.

Finally, notice that the high degree of interpretability of PID and our simple task setups allowed for a very intuitive reasoning about hyperparameters with only minor fine-tuning. However, more complex tasks with bigger infomorphic networks might require more systematic hyperparameter optimization techniques, a variety of which are easily accessible in modern-day machine learning tools (83). Fortunately, the resulting hyperparameter sets will then still be formulated in the language of Partial Information Decomposition and thus potentially provide crucial insights into the optimal local goals to enable the self-organization and collaboration of local units to solve a variety of global tasks (77).

### 4.2. Conclusion.

Leveraging Partial Information Decomposition, this work establishes the infomorphic neuron, a neuron model permitting the flexible and direct optimization of interpretable information-theoretic goals. Through several lines of experimentation, the versatility of these neurons to solve a variety of machine learning tasks has been demonstrated. We propose infomorphic neurons as abstract neuron models that can provide a foundation for studying information processing in neural networks in the language of local goals, opening up many exciting avenues for future research.

## 5. Materials and Methods

Here, we provide the material and methods that apply to all our simulation experiments, while specific variations are discussed in the respective subsections of Section 3. In all simulation experiments, we run discrete-time neural networks. After choosing the network topology, the goal function, and activation function of each neuron, we initialize all weights at small values. Stimuli vary by experiment and are always presented to the networks sequentially, without reinitialization of neural activities. For each experiment, we segment the execution time into minibatches of fixed length. After each minibatch, we construct the full binned probability distribution $p_{Y,\tilde{R},\tilde{C}}$ of each neuron from the histogram of its inputs and outputs, which are obtained by quantizing R and C in uniform bins. Assuming the histogram as constant, we compute the gradients of the information-theoretic neural goal function G with respect to the weights according to Eqs. **8** and **9**, and conduct a weight update by applying gradient descent with a fixed learning rate.

To evaluate each experiment, we calculate performance metrics over the course of training by interleaving the training data stream with test data at regular intervals, while suppressing weight updates. These metrics include both the “information dynamics,” i.e., the size of all information atoms of each neuron, and more traditional performance metrics like task accuracy, which our algorithm does not explicitly optimize for. For details on the parameter choices and results of each experiment, we refer the reader to the respective experimental Sections 3.1, 3.2, and 3.3 and to *SI Appendix*, sections 2 and 3. The experiments have been implemented in Python and have been made available on GitLab at https://gitlab.gwdg.de/wibral/infomorphic_networks (84).

## Supplementary Material

Appendix 01 (PDF)

## Data Availability

The full derivation of the learning rules, parameters for all experiments, as well as supplementary figures providing additional information on the experiments are provided in *SI Appendix*. Code for reproducing all experiments is available on GitLab at https://gitlab.gwdg.de/wibral/infomorphic_networks (84). The raw data of the experiments are accessible via Göttingen Research Online Data at https://doi.org/10.25625/0M1PTJ (85). All other data are included in the manuscript and/or *SI Appendix*.

## Footnotes

^*^Recently, PID has also been used to describe the function of cortical neurons (47) and the representation of information in artificial and biological neural networks (48–51).
