# Supplementary material for: A general framework for interpretable neural learning based on local information-theoretic goal functions
Source: Proc Natl Acad Sci U S A. 2025 Mar 5;122(10):e2408125122. doi: 10.1073/pnas.2408125122 (PMC11912414; doi:10.1073/pnas.2408125122)
Supplement: Supplementary file 1 — Appendix 01 (PDF) [file pnas.2408125122.sapp.pdf]

Supporting Information for

A General Framework for Interpretable Neural Learning based on Local Information-Theoretic Goal Functions

Abdullah Makkeh, Marcel Graetz, Andreas C. Schneider, David A. Ehrlich, Viola Priesemann and Michael Wibral

Abdullah Makkeh.
E-mail: abdullah.alimakkeh@uni-goettingen.de;
Marcel Graetz.
E-mail: marcel.graetz@research.fchampalimaud.org;
Andreas Schneider.
E-mail: andreas.schneider@ds.mpg.de;
Michael Wibral.
E-mail:, michael.wibral@uni-goettingen.de

This PDF file includes:

Figs. S1 to S10
Tables S1 to S4
SI References

1. Derivation of the learning rules

In the following we provide a detailed analytical derivation of the learning rules.

The value of the goal function is dependent on the neuron-specific parameter vector $\mathbf{\Gamma}$ and the joint distribution $p(R, C, Y)$ of a neuron's integrated receptive and contextual inputs and its output. Learning happens by changing the afferent weights $\mathbf{w}_R$ and $\mathbf{w}_C$ which determine how the inputs to the neuron influence its output. To learn these weights via gradient descent, we require the gradients $\frac{\partial G}{\partial \mathbf{w}_R}$ and $\frac{\partial G}{\partial \mathbf{w}_C}$. Starting from the definition of G, we first make use of the fact that a computable expression for one PID atom, in this case the redundant information $I_{\text{red}}(Y : R, C) := I_\cap^{\text{sx}}(Y : R; C)$, in combination with the rules of classical information theory suffices to quantify all four PID atoms (1). Note that we stick to the notation ";" in $I_c^{\text{sx}}ap$ introduced by Makkeh et al. (2) which stands for the union of R and C stressing that $I_\cap^{\text{sx}}$ is based on union-exclusions. This allows us to re-parametrize

$$
\begin{aligned}
G(Y : R, C) &= \Gamma_{\text{unq},R} I_{\text{unq},R}(Y : R, C) + \Gamma_{\text{unq},C} I_{\text{unq},C}(Y : R, C) + \Gamma_{\text{red}} I_{\text{red}}(Y : R, C) + \Gamma_{\text{syn}} I_{\text{syn}}(Y : R, C) + \Gamma_{\text{res}} H(Y \mid R, C) \\
&=: \gamma_Y H(Y) + \gamma_{Y|R} H(Y \mid R) + \gamma_{Y|C} H(Y \mid C) + \gamma_{Y|R,C} H(Y \mid R, C) + \gamma_{\text{red}} I_{\text{red}}(Y : R, C) \;,
\end{aligned}
$$

with the parameter transformation

$$
\mathbf{\Gamma} = \begin{pmatrix} \Gamma_{\text{unq},R} \\ \Gamma_{\text{unq},C} \\ \Gamma_{\text{red}} \\ \Gamma_{\text{syn}} \\ \Gamma_{\text{res}} \end{pmatrix} =: \begin{pmatrix} 1 & 0 & 1 & 0 & 0 \\ 1 & 1 & 0 & 0 & 0 \\ 1 & 0 & 0 & 0 & 1 \\ 1 & 1 & 1 & 0 & 0 \\ 1 & 1 & 1 & 1 & 0 \end{pmatrix} \begin{pmatrix} \gamma_Y \\ \gamma_{Y|R} \\ \gamma_{Y|C} \\ \gamma_{Y|R,C} \\ \gamma_{\text{red}} \end{pmatrix} \quad \Longleftrightarrow \quad \gamma := \begin{pmatrix} \gamma_Y \\ \gamma_{Y|R} \\ \gamma_{Y|C} \\ \gamma_{Y|R,C} \\ \gamma_{\text{red}} \end{pmatrix} := \begin{pmatrix} 1 & 1 & 0 & -1 & 0 \\ -1 & 0 & 0 & 1 & 0 \\ 0 & -1 & 0 & 1 & 0 \\ 0 & 0 & 0 & -1 & 1 \\ -1 & -1 & 1 & 1 & 0 \end{pmatrix} \begin{pmatrix} \Gamma_{\text{unq},R} \\ \Gamma_{\text{unq},C} \\ \Gamma_{\text{red}} \\ \Gamma_{\text{syn}} \\ \Gamma_{\text{res}} \end{pmatrix} .
$$

Next, notice that G is only dependent on the weights through the conditional firing probability of the neuron $\theta(r, c) = P(Y = +1 \mid R = r, C = c)$. Ultimately, we want to arrive at an expression of the form

$$
\begin{aligned}
\frac{\partial G}{\partial \mathbf{w}_R} &= \left\langle \frac{\partial g(r,c)}{\partial \theta(r,c)} \frac{\partial \theta(r,c)}{\partial \mathbf{w}_R} \right\rangle_{r,c} \\
\frac{\partial G}{\partial \mathbf{w}_C} &= \left\langle \frac{\partial g(r,c)}{\partial \theta(r,c)} \frac{\partial \theta(r,c)}{\partial \mathbf{w}_C} \right\rangle_{r,c} ,
\end{aligned}
$$

where we expressed G as an expectation over the joint distribution of the integrated inputs $p(r, c)$. Introducing a quantity g that we can express for every value of r and c allows us to then apply the chain rule, and is possible due to the fact that all information-theoretic terms in G are point-wise measures, including the redundant information measure (2)

$$
I_{\text{red}}(Y : R, C) = I_\cap^{\text{sx}}(Y : R; C) = \langle i_\cap^{\text{sx}}(y : r; c) \rangle_{r,c,y} := \left\langle \log \frac{p_\theta(y \mid r \cup c)}{p_\theta(y)} \right\rangle_{r,c,y} ,
$$

where we have explicitly marked θ-dependence by a subscript, and introduced the term

$$
p_\theta(y \mid r \cup c) = \frac{p_\theta(y, r \cup c)}{p(r \cup c)}
$$

with

$$
\begin{aligned}
p(r \cup c) &= p(r) + p(c) - p(r, c) \\
p_\theta(y, r \cup c) &= p(y, r) + p(y, c) - p(y, r, c) \\
&= \left\langle \theta(r, c') p(r, c') \right\rangle_{c'} + \left\langle \theta(r', c) p(r', c) \right\rangle_{r'} - \theta(r, c) p(r, c).
\end{aligned}
$$

Inserting definitions and separating the terms for $y = 1$ and $y = -1$, we can now write

$$
\begin{aligned}
G(Y:R,C) = &-\gamma_Y \Big[\, p(+1)\log p(+1) + p(-1)\log p(-1) \,\Big] \\
&-\gamma_{Y|R} \Big\langle\, p(+1|r)\log p(+1|r) + p(-1|r)\log p(-1|r) \,\Big\rangle_r \\
&-\gamma_{Y|C} \Big\langle\, p(+1|c)\log p(+1|c) + p(-1|c)\log p(-1|c) \,\Big\rangle_c \\
&-\gamma_{Y|R,C} \Big\langle\, p(+1|r,c)\log p(+1|r,c) + p(-1|r,c)\log p(-1|r,c) \,\Big\rangle_{r,c} \\
&-\gamma_{\text{red}} \Big[\, p(+1)\log p(+1) + p(-1)\log p(-1) \,\Big] \\
&+\gamma_{\text{red}} \Big\langle\, p(+1|r,c)\log p(+1|r\cup c) + p(-1|r,c)\log p(-1|r\cup c) \,\Big\rangle_{r,c} \\
= &-\gamma_Y \Big\langle\, \theta \log\langle\theta\rangle_{r,c} + (1-\theta)\log(1-\langle\theta\rangle_{r,c}) \,\Big\rangle_{r,c} \\
&-\gamma_{Y|R} \Big\langle\, \theta \log\langle\theta\rangle_{c|r} + (1-\theta)\log(1-\langle\theta\rangle_{c|r}) \,\Big\rangle_{r,c} \\
&-\gamma_{Y|C} \Big\langle\, \theta \log\langle\theta\rangle_{r|c} + (1-\theta)\log(1-\langle\theta\rangle_{r|c}) \,\Big\rangle_{r,c} \\
&-\gamma_{Y|R,C} \Big\langle\, \theta \log\theta + (1-\theta)\log(1-\theta) \,\Big\rangle_{r,c} \\
&-\gamma_{\text{red}} \Big\langle\, \theta \log\langle\theta\rangle_{r,c} + (1-\theta)\log(1-\langle\theta\rangle_{r,c}) \,\Big\rangle_{r,c} \\
&+\gamma_{\text{red}} \Big\langle\, \theta \log p_\theta(+1|r\cup c) + (1-\theta)\log(1-p_\theta(+1|r\cup c)) \,\Big\rangle_{r,c} ,
\end{aligned}
$$

where in the second equation we have made the dependence on θ explicit and written all terms in the same expectation value. From the first equation, it is straightforward to see that differentiating the logarithms leads to terms that cancel in all cases except the last line of the redundant information. Taking into account that

$$
\begin{aligned}
\theta(r,c) &= \sigma(A(r,c)) = \sigma(A(\mathbf{w}_R^T\mathbf{x}_R, \mathbf{w}_C^T\mathbf{x}_C)) \\
\Rightarrow \frac{\partial\theta(r,c)}{\partial\mathbf{w}_R} &= \theta(r,c)(1-\theta(r,c))\frac{\partial A}{\partial r}\mathbf{x}_R \\
\frac{\partial\theta(r,c)}{\partial\mathbf{w}_C} &= \theta(r,c)(1-\theta(r,c))\frac{\partial A}{\partial c}\mathbf{x}_C ,
\end{aligned}
$$

with the sigmoid function $\sigma(\xi) = \frac{1}{1+e^{-\xi}}$; $\frac{d\sigma}{d\xi} = \sigma(\xi)(1-\sigma(\xi))$, we thus obtain

$$
\begin{aligned}
\frac{\partial G}{\partial\mathbf{w}_R} &= \left\langle \frac{\partial g(r,c)}{\partial\theta(r,c)}\theta(r,c)(1-\theta(r,c))\frac{\partial A}{\partial r}\mathbf{x}_R \right\rangle_{r,c} \\
\frac{\partial G}{\partial\mathbf{w}_C} &= \left\langle \frac{\partial g(r,c)}{\partial\theta(r,c)}\theta(r,c)(1-\theta(r,c))\frac{\partial A}{\partial c}\mathbf{x}_C \right\rangle_{r,c}
\end{aligned}
$$

where

$$
\begin{aligned}
\frac{\partial g(r,c)}{\partial\theta(r,c)} = &-(\gamma_Y + \gamma_{\text{red}})\log\frac{\langle\theta\rangle_{r,c}}{1-\langle\theta\rangle_{r,c}} - \gamma_{Y|R}\log\frac{\langle\theta\rangle_{c|r}}{1-\langle\theta\rangle_{c|r}} - \gamma_{Y|C}\log\frac{\langle\theta\rangle_{r|c}}{1-\langle\theta\rangle_{r|c}} + \gamma_{Y|R,C}A(r,c) \\
&+ \gamma_{\text{red}}\left[\log\left(\frac{p_\theta(+1|r\cup c)}{1-p_\theta(+1|r\cup c)}\right) + \left(\frac{\theta}{p_\theta(+1|r\cup c)} - \frac{1-\theta}{1-p_\theta(+1|r\cup c)}\right)p(r\cup c)\right].
\end{aligned}
$$

Taking the expectation values over $p(r,c)$ instead of $p(\mathbf{x}_R, \mathbf{x}_C)$ here does not change the outcome when working with the empirically sampled inputs, but significantly simplifies notation and calculations (3). Note that $\frac{\partial G}{\partial\mathbf{w}_R}$ and $\frac{\partial G}{\partial\mathbf{w}_C}$ inherit the term $\theta(r,c)(1-\theta(r,c))$ from the derivative of the sigmoid function. This term is small whenever the firing probability is far from $\theta(r,c) = \frac{1}{2}$, and gives inputs that lead to highly stochastic firing increased influence on learning.

To arrive at the empirical gradients, we substitute the expectation value over the joint distribution of the integrated inputs by an empirical average over the t-th batch and obtain

$$
\begin{aligned}
\frac{\partial\hat{G}^t}{\partial\mathbf{w}_R} &= \frac{1}{|B^t|}\sum_{\{\mathbf{x}_R,\mathbf{x}_C\}\in B^t}\left[\left.\frac{\partial g}{\partial\theta}\right|_{\tilde r,\tilde c}\theta(\tilde r,\tilde c)(1-\theta(\tilde r,\tilde c))\left.\frac{\partial A}{\partial r}\right|_{\tilde r,\tilde c}\mathbf{x}_R\right] \\
&:= \frac{1}{|B^t|}\sum_{\{\mathbf{x}_R,\mathbf{x}_C\}\in B^t}\left[f^{\mathbf{\Gamma}}_{p(R,C)}(\tilde r,\tilde c)\left.\frac{\partial A}{\partial r}\right|_{\tilde r,\tilde c}\mathbf{x}_R\right] \\
\frac{\partial\hat{G}^t}{\partial\mathbf{w}_C} &= \frac{1}{|B^t|}\sum_{\{\mathbf{x}_R,\mathbf{x}_C\}\in B^t}\left[\left.\frac{\partial g}{\partial\theta}\right|_{\tilde r,\tilde c}\theta(\tilde r,\tilde c)(1-\theta(\tilde r,\tilde c))\left.\frac{\partial A}{\partial c}\right|_{\tilde r,\tilde c}\mathbf{x}_C\right] \\
&:= \frac{1}{|B^t|}\sum_{\{\mathbf{x}_R,\mathbf{x}_C\}\in B^t}\left[f^{\mathbf{\Gamma}}_{p(R,C)}(\tilde r,\tilde c)\left.\frac{\partial A}{\partial c}\right|_{\tilde r,\tilde c}\mathbf{x}_C\right] .
\end{aligned}
$$

where B^t denotes the batch corresponding to training time t, and the batch size $|B^t| = N_{\text{tr}}$ or $|B^t| = N_{\text{te}}$ is constant in all our experiments. Furthermore, $(\tilde{r}, \tilde{c})$ are the binned observations, and we use $h(\tilde{r}, \tilde{c})$ as a shorthand to indicate that we are evaluating any function h at the bin center of the bin corresponding to the tuple (r, c). The notation with $f^{\mathbf{\Gamma}}_{p(R,C)}(\tilde{r}, \tilde{c})$ corresponds to equations [8] and [9] in the main manuscript.

Finally, this leads to the update rules

$$\mathbf{w}_R^{t+1} = \mathbf{w}_R^t + \eta \frac{\partial \hat{G}^t}{\partial \mathbf{w}_R}$$
$$\mathbf{w}_C^{t+1} = \mathbf{w}_C^t + \eta \frac{\partial \hat{G}^t}{\partial \mathbf{w}_C} \ .$$

2. Experiments: parameters and statistics

In the following we will give a full account of the setup and parameter choices of the experiments that will allow reproduction of our results. We will first explain each parameter and then tabulate the parameter values for all three experimental schemes (supervised, unsupervised and memory).

A. Explanation of parameters. Each infomorphic neuron has a set of parameters that determine its function and goal. Additionally, in each task, the training of these neurons might slightly vary. The training and individual neuron parameters are:

- **Training:**
  - phases: In the unsupervised learning experiment, neurons undergo training in two phases, each with different parameter settings. We provide the number of training steps for each phase as a tuple of integers in the "phases" parameter. In the other experiments, this parameter is simply an integer indicating the number of training steps.
  - N_{tr} and N_{te}: The number of training and testing samples in a mini-batch per training step, respectively.
  - m_{rep}: The number of times an individual input is presented. We introduce this parameter as the unsupervised and memory experiments require presentation of each input over multiple time steps.
- **Learning:**
  - b_{init}: The order of magnitude of the initialized weights. For example $b_{\text{init}} = 0.1$ means that the weights are initialized uniformly at random in the interval $[-0.1, 0.1]$.
  - goal function parameters Γ: The parameters defining which information contributions a neuron maximizes or minimizes. We use the vector notation $\mathbf{\Gamma} = (\Gamma_{\text{unq},R}, \Gamma_{\text{unq},C}, \Gamma_{\text{red}}, \Gamma_{\text{syn}}, \Gamma_{\text{res}})$. For example, in the supervised learning experiment, $\Gamma = (0.1, 0.1, 1, 0.1, 0)$. The parameter vector $\mathbf{\Gamma}$ does not need to be of unit length and deviations from unit length will effectively act as a scaling factor on the learning rate.
  - learning rate η: The learning rate for the gradient ascent utilized to maximize the goal function.
  - pullback rate λ: The weights decay factor. In the first training phase of the unsupervised learning experiment, the receptive weights are pulled back by λ at each step to keep them from growing. This pullback acts as follows: $w_R = w_R - 2\lambda w_R + \eta \nabla(w_R)$ where $\nabla(w_R)$ is the gradient update at this step.
- **Input Integration:**
  - $n_{\text{receptive}}$: The dimension of the input vector $\mathbf{X}_R$, i.e. the number of receptive inputs. Evidently, $n_{\text{receptive}}$ is also the size of the vector $\mathbf{w}_R$.
  - $n_{\text{contextual}}$: The dimension of the input vector $\mathbf{X}_C$, i.e. the number of contextual inputs. Evidently, $n_{\text{contextual}}$ is also the size of the vector $\mathbf{w}_C$.
  - J_R and J_C: The interval in which the R and C values are binned, respectively. Any realizations r or c exceeding the limits of their respective interval are aggregated into two additional bins of infinite width at either side.
  - $n_{\text{bins},r}$ and $n_{\text{bins},c}$: The number of equal-width bins in the intervals J_R and J_C, respectively. The total number of bins is $(n_{\text{bins},r} + 2) \times (n_{\text{bins},c} + 2)$ due to the bins of infinite width bounding the respective intervals. The discrete random variables resulting from the binning of R and C are denoted as $\tilde{R}$ and $\tilde{C}$.

B. Supervised learning. In this experiment 100 networks were run. All networks were identical in architecture, with a single layer of 10 neurons, but initialized at different sets of random weights. During training, the 28-by-28 MNIST pixel images were presented in random order as the 784-dimensional vector $\mathbf{X}_R$, with each pixel value rescaled to the interval $[-1, 1]$. The label was presented as a one-hot representation $\mathbf{X}_C$. During testing, the network received the MNIST image as in training, however, a constant vector of zeroes on $\mathbf{X}_C$. Note that during training $X_C \in -1, 1$, so a value of $X_C = 0$ is equidistant from both possible values during training. The training and individual neuron parameters are summarized in Table S1.

Table S1. ***Parameters of the infomorphic neurons in the supervised learning experimental scheme.***

Training		
Parameter	Value	Comment
Phases	800	a single phase of training with 800 training steps (batches)
N_{tr}	1000	batch sampled uniformly from the 60000 MNIST training images
N_{te}	1000	batch sampled uniformly from the 10000 MNIST testing images
m_{rep}	1	no repetition is needed
**Learning**		
Parameter	Value	Comment
b_{init}	0.01	scale of initialization
$\mathbf{\Gamma}$	(0.1, 0.1, 1, 0.1, 0)	goal parameters ($\Gamma_{\mathrm{unq},R}$, $\Gamma_{\mathrm{unq},C}$, Γ_{red}, Γ_{syn}, Γ_{res})
η	1.0	learning rate
λ	0.0	no pullback needed
**Input Integration**		
Parameter	Value	Comment
$n_{\mathrm{receptive}}$	784	MNIST image pixel size
$n_{\mathrm{contextual}}$	1	one element of a one-hot label vector
J_R	$[-20, 20]$	a smaller range might hinder the learning
J_C	$[-20, 20]$	a smaller range might hinder the learning
n_R-bins	200	uniform bin-size is 0.1
n_C-bins	200	uniform bin-size is 0.1

C. Unsupervised Learning. In this experiment 300 networks were run. All networks were identical in architecture with a single layer of 8 neurons, but initialized at different sets of random weights. The neurons were recurrently connected via their contextual inputs $\mathbf{x}_C$. During training and testing, an 8-by-8 grid of pixels was received as the 64-dimensional vector $\mathbf{X}_R$ with discrete pixel values in $\{-1, 1\}$. Training was split into two phases: the first with a weight decay to keep the weights low and allow communication between the neurons, while in the second phase the weight decay was turned off to make the magnitude of all weights increase to the final solution. The training and individual neuron parameters are summarized in Table S2.

Table S2. ***Parameters of the infomorphic neurons in the unsupervised learning experimental scheme.***

Training		
Parameter	Value	Comment
Phases	$(50, 50)$	two phases of training with 50 training steps (batches) each, first with weight decay activated
N_{tr}	1000	batch generated randomly from a distribution of independent bars with $p = 0.5$ for each bar
N_{te}	1000	batch generated randomly from a distribution of independent bars with $p = 0.5$ for each bar
m_{rep}	8	each sample is presented 8 consecutive time steps
**Learning**		
Parameter	Value	Comment
b_{init}	0.1	scale of initialization
$\mathbf{\Gamma}$	(1, 0, 0, 0, 0)	goal parameters ($\Gamma_{\mathrm{unq},R}$, $\Gamma_{\mathrm{unq},C}$, Γ_{red}, Γ_{syn}, Γ_{res}), equal in both learning phases
η	(10.0, 1.0)	higher learning rate during weight decay phase
λ	(0.28, 0.0)	initial phase of weight decay
**Input Integration**		
Parameter	Value	Comment
$n_{\mathrm{receptive}}$	64	size of the input
$n_{\mathrm{contextual}}$	7	one per each neuron with no self connections
J_R	$[-25, 25]$	a smaller range might hinder the learning
J_C	$[-25, 25]$	a smaller range might hinder the learning
n_R-bins	500	uniform bin-size is 0.05; larger size might hinder the learning
n_C-bins	500	uniform bin-size is 0.05; larger size might hinder the learning

D. Associative Memory. In this experiment 425 individual networks of 100 neurons were run. The networks are clustered into 17 groups of 25 networks each; each of which was trained on a different number of memory patterns in $\{1, 2, 4, 6, 8, 12, 15, 20, 25, 30, 35, 40, 45, 50, 55, 60\}$. All memory patterns were chosen as random 100-dimensional binary vectors with exactly 50 entries being

+1 and 50 entries being −1, indicated as ($p = 0.5$)-sparsity in the main text. All networks were identical in architecture with a single layer of 100 neurons, but initialized at different sets of random weights. The neurons were recurrently connected via their contextual weights $\mathbf{w}_C$, excluding self-connections. During training, each neuron received a single input $x_R \in \{-1, 1\}$ at every time step, i.e. one bit of the presented memory pattern. During testing (recall), the pattern was presented only in the first time step, then for later steps x_R was set to 0 for all neurons. For each of the 19 consecutive time steps of the recall, the responses of the neurons were read out and compared with the originally presented pattern using cosine similarity. In the main text we report the similarity in the last recall time step as the accuracy. The training and individual neuron parameters are summarized in Table S3.

Table S3. ***Parameters of the infomorphic neurons in the associative memory experimental scheme.***

Training		
Parameter	Value	Comment
Phases	300	a single phase of training with 300 epochs
N_{tr}	200	sampled from the set of input patterns
N_{te}	200	sampled from the set of input patterns
m_{rep}	8	each sample is presented 8 consecutive times
**Learning**		
Parameter	Value	Comment
b_{init}	0.1	scale of initialization
$\mathbf{\Gamma}$	(0.1, 0.1, 1, 0.1, 0)	goal parameters $(\Gamma_{\mathrm{unq},R}, \Gamma_{\mathrm{unq},C}, \Gamma_{\mathrm{red}}, \Gamma_{\mathrm{syn}}, \Gamma_{\mathrm{res}})$
η	0.48	$\eta = 0.5$ does not change the result
λ	0.0	no weight pullback
**Input Integration**		
Parameter	Value	Comment
$n_{\mathrm{receptive}}$	1	bit of the presented pattern
$n_{\mathrm{contextual}}$	99	a one per each neuron with no self connections
J_R	$[-20, 20]$	a smaller range might hinder the learning
J_C	$[-20, 20]$	a smaller range might hinder the learning
n_R-bins	20	uniform bin-size is 2; smaller size doesn't affect the learning
n_C-bins	20	uniform bin-size is 2; smaller size doesn't affect the learning

3. Experiments: Supplementary Observables

In this section we show observables that complement the main observables shown for each experiment in the main paper. These observables are meant to give a more in depth account for various performance measures of the infomorphic networks in the supervised, unsupervised, and memory tasks.

A. Supervised learning. Figure S1 shows the local information contributions of all ten neurons from one randomly chosen network, the respective confusion matrix and the averaged firing probability of each neuron for each label. High sensitivity and specificity of a neuron to its assigned digit correspond to high redundant information I_{red} and low values of all other information contributions (e.g. neurons 0, 1, and 6). In particular, in these cases both the entropy of the neuron and its redundant information approach the entropy of the respective label in the test data set. High firing probability of a neuron for digits from the wrong class (false positives) corresponds to a reduction of its redundant information I_{red} and an increase of the unique information of its receptive inputs $I_{\mathrm{unq},R}$ (e.g. neurons 5, 8, and 9). Lower firing probability for the correct digit (fewer true positives) without high firing probability for wrong digits (few false positives) results in a weaker decrease of redundant and increase of unique information (e.g. neurons 2, 3, 4, 7).

To compare learning outcome and accuracy of the infomorphic networks, we additionally trained a logistic regression model on one-vs-all classification of MNIST digits for 3000 iterations with vanilla gradient descent and a learning rate of $\eta = 1$ (adapted from (4)).

Figure S2 compares the receptive fields of the infomorphic neurons to those obtained from logistic regression. Overall, we find a high degree of cosine similarity. Furthermore, visual inspection as well as the cosine similarity for each pair of corresponding rows and columns indicate that the receptive fields are particularly similar in the center of each image that contains the pixels that are relevant for classification Figure S2. Meanwhile, differences between the receptive fields are most pronounced closer to the image borders, with infomorphic networks seemingly utilizing these non-coding pixels as an additional bias term.

Figure S3 compares two approaches of computing the test accuracy: the *absent-label approach* where the test accuracy is the winner take all accuracy $P(Y|r, 0)$, i.e. $x_c = 0$ during testing and the *marginalized-label approach* where the test accuracy is the winner take all accuracy using $P(Y|r) \approx \sum_{c_{\mathrm{train}}} P(Y|r, c_{\mathrm{train}})P(c_{\mathrm{train}})$, i.e., c is provided but marginalized using the prior probabilities $P(c_{\mathrm{train}})$ from the training set during testing. Thus, in different ways, both approaches hide the information of

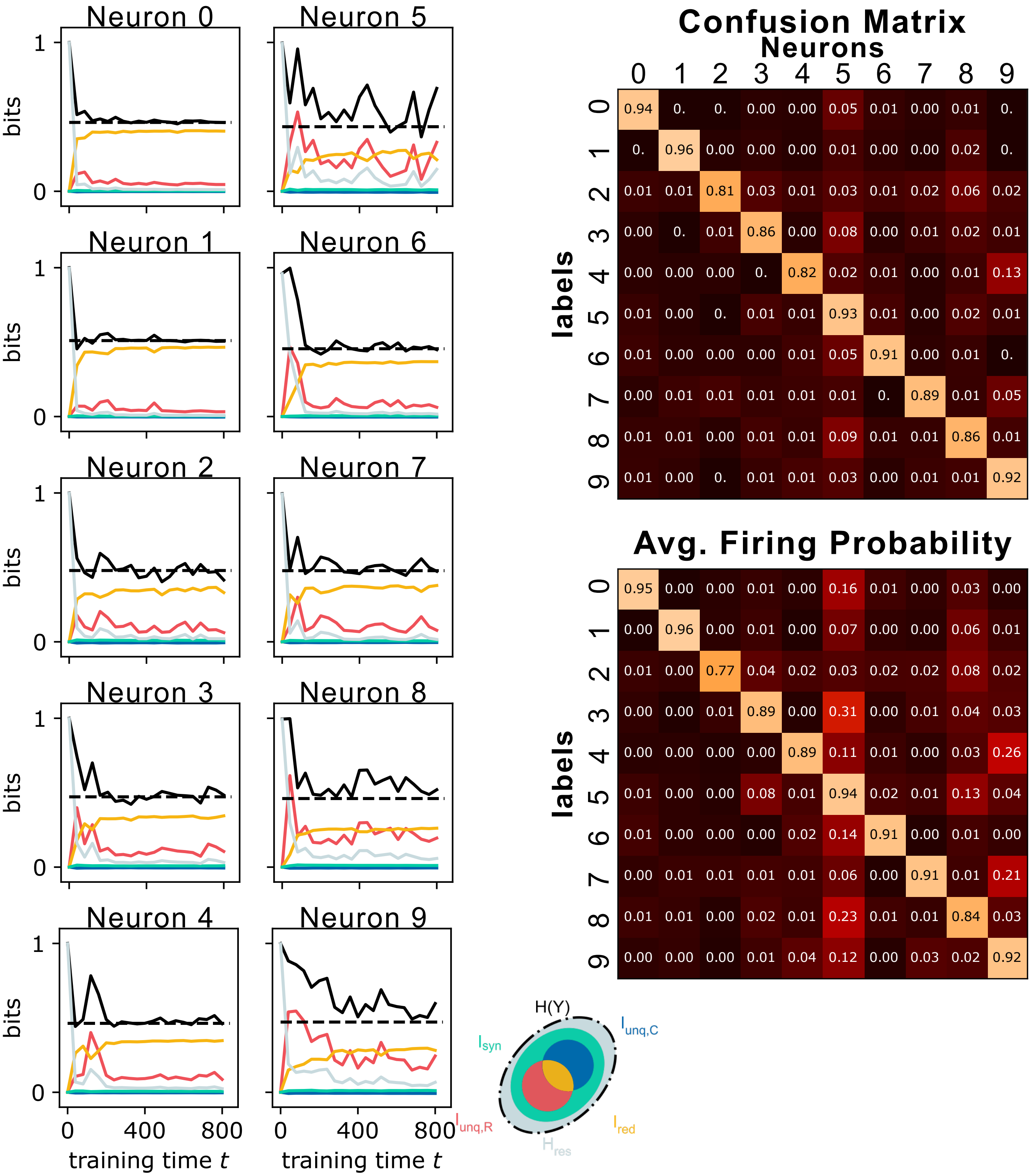

Fig. S1. In supervised learning, high redundant information I_{red} and low unique receptive information $I_{\mathrm{unq},R}$ are associated with high classification accuracy. (Left) Time evolution of information quantities for each neuron in a single, randomly chosen network performing supervised learning. Neuron i corresponds to label i. The dashed line shows the empirical entropy of the binary one-vs-all distribution of each label in the test data set. **(Top right)** The confusion matrix for this network, after applying a winner-take-all readout. **(Bottom right)** The firing probability of each neuron for each digit, averaged over test images.

the actual contextual input (teacher signal) from the neuron while making the decision. Empirically, both approaches yield indistinguishable accuracy, highlighting that contextual inputs only have a modulating role, in line with our choice of activation function. Importantly, however, only the first approach is plausible in actual neural networks, as neurons cannot marginalize

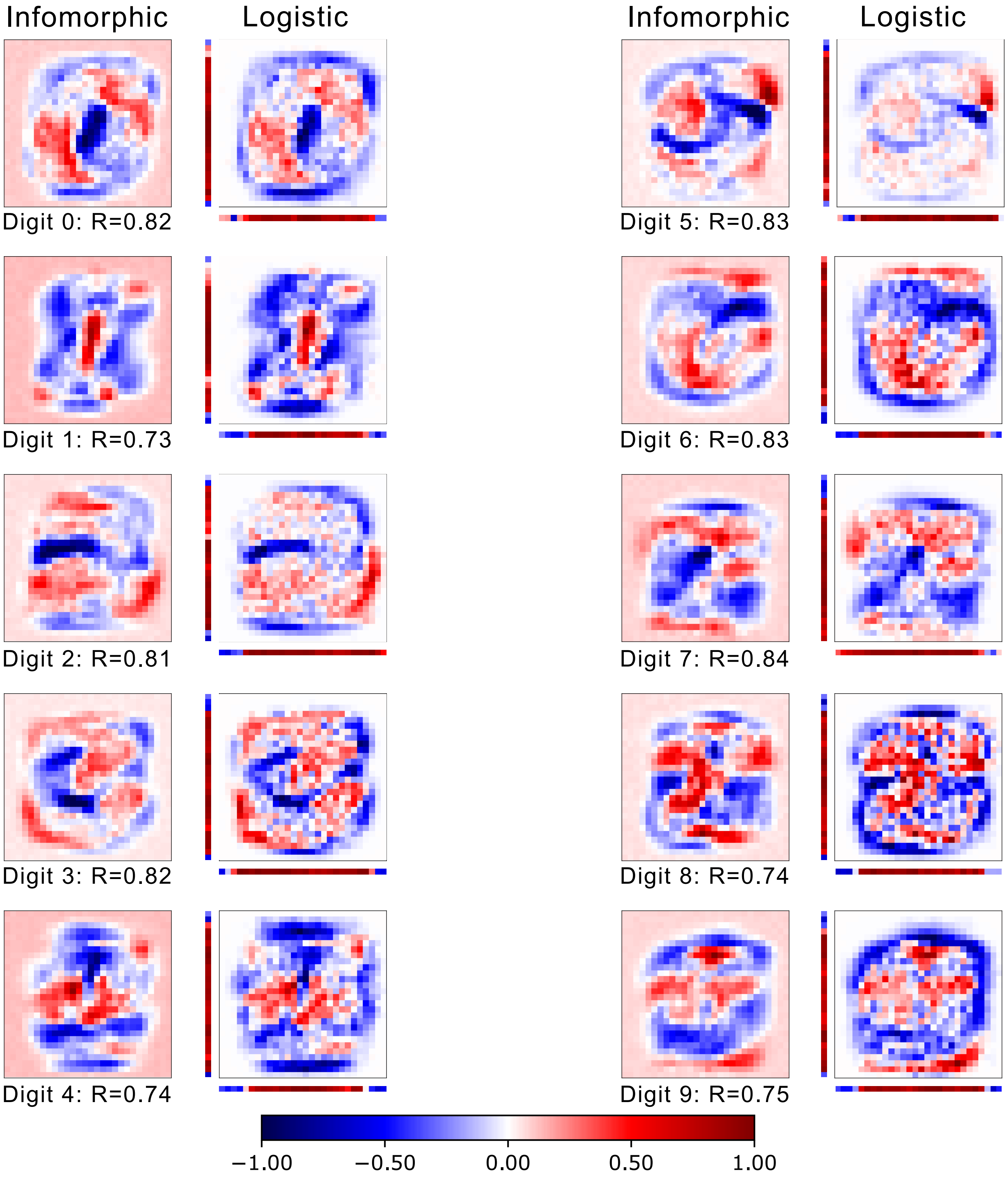

Fig. S2. The receptive fields of supervised infomorphic learning are similar to those of one-vs-all logistic regression. First and third column: Receptive fields of all neurons after training in a randomly chosen supervised infomorphic network. Second and fourth column: Corresponding receptive fields obtained from one-vs-all logistic regression with vanilla gradient descent. The depicted receptive fields are centered at a weight of $w_R = 0$ and re-scaled to the interval $[-1, 1]$. R-values indicate cosine similarity between corresponding receptive fields. The colored vectors bordering the receptive fields of logistic regression indicate row-by-row and column-by-column cosine similarity between corresponding receptive fields. Note that all values are on the same scale, indicated by the color bar at the bottom.

over their inputs before calculating their output.

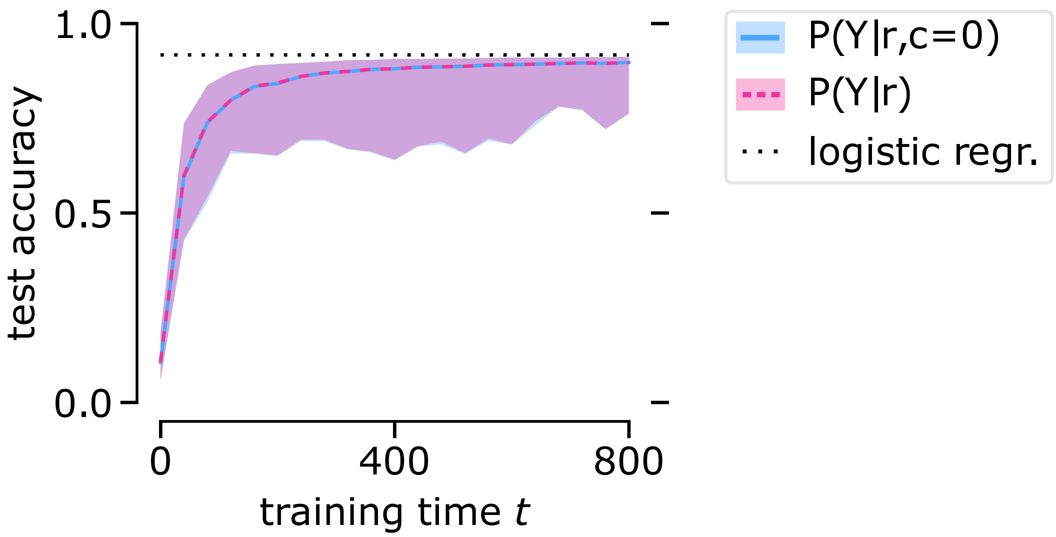

Fig. S3. The test accuracy of supervised infomorphic learning is similar using $P(Y \mid r, 0)$ or $P(Y \mid r)$. The average winner-take-all test accuracy using $P(Y|r, 0)$ and $P(Y|r)$ across 100 training runs, with both accuracies approaching that of logistic regression (reaching on average 89.7% vs. 91.9% for log. regr.). Note that the 95-percentile is being displayed.

B. Unsupervised learning. Figure S4 shows the evolution of the local information contributions of all the eight neurons over the course of training, and their receptive and contextual fields after training, from one randomly chosen network out of 298 networks that successfully encoded all eight bars. For the same network, Figure S5 shows the evolution of the receptive weights of all neurons, and Figure S6 shows the evolution of the contextual weights. It is evident from these three figures that all relevant learning happens in the first phase of training (specifically the first 20 mini-batches) which includes a weight decay on the receptive weights after every training step. Due to the weight decay, all neurons have overall low receptive weights, meaning their firing is highly stochastic. In this phase, neurons are competing for the encoding of individual bars, as seen from their receptive weights (Figure S5), until all neurons have settled on an individual bar. This happens because coding for the same bars introduces redundant information I_{red}, which neurons need to reduce in order to maximize their unique receptive information $I_{\text{unq},R}$, as by definition $I_{\text{red}} + I_{\text{unq},R} = I(Y : R) \leq 1$.

In the second training phase, neurons mainly increase the receptive weight of their preferred bar, reducing their stochasticity and thereby increasing their unique information further. To our surprise, in this phase the contextual weights stay almost constant, an effect for which we currently have no intuitive explanation. Due to these constant and often non-zero contextual weights, each neuron is influenced to varying degrees by the other neurons. This influence leads each neuron to partly code for the bars of other neurons, despite our choice of an activation function that predominantly depends on the receptive input. This indirect encoding of bars other than the preferred one introduces a certain degree of redundant information I_{red}, as the information about these non-preferred bars is also contained in the receptive input, i.e. the image. Interestingly, most neurons learn to compensate for this indirect influence by learning receptive weights for the respective bars that counteract the effect of these contextual inputs on their output. It is for this reason that the receptive fields of most neurons show additional weak traces of bars other than their preferred bar (Figure S4).

Figure S7 shows the evolution of the local information contributions of all the eight neurons over the course of training, and their receptive and contextual fields after training, from one randomly chosen network out of 2 unsuccessful networks. Here, the first and the last neuron converged onto the same bar, while the last bar is not encoded by any neuron. As a result, both neurons fail to increase their unique receptive information $I_{\text{unq},R}$ and instead exhibit high redundant information I_{red} after training.

Figure S8 shows a summary of the receptive weights from all 300 randomly initialized runs, out of which 298 led to each neuron encoding a separate bar, and 2 led to an encoding of only seven out of eight bars, with one redundantly encoded bar as shown in Figure S7.

C. Associative memory. Figure S9 shows the local information contributions averaged over all neurons, for 25 networks trained on 4, 12, 35, 60 patterns each. As mentioned in the main text, infomorphic networks cannot reliably encode very few (e.g. 4) patterns, as in this case $\frac{100}{2^3} = 12.5$ neurons are expected to receive the exact same receptive input for every pattern, resulting in $I(Y : R) = 0$ bits of information in their receptive inputs. Concurrently, the average redundant information I_{red} is comparably low and the unique contextual information $I_{\text{unq},C}$ comparably high in these networks, indicating that the recurrent weights of certain neurons are not aligned with their external input, but instead retain unique information induced by their random initialization.

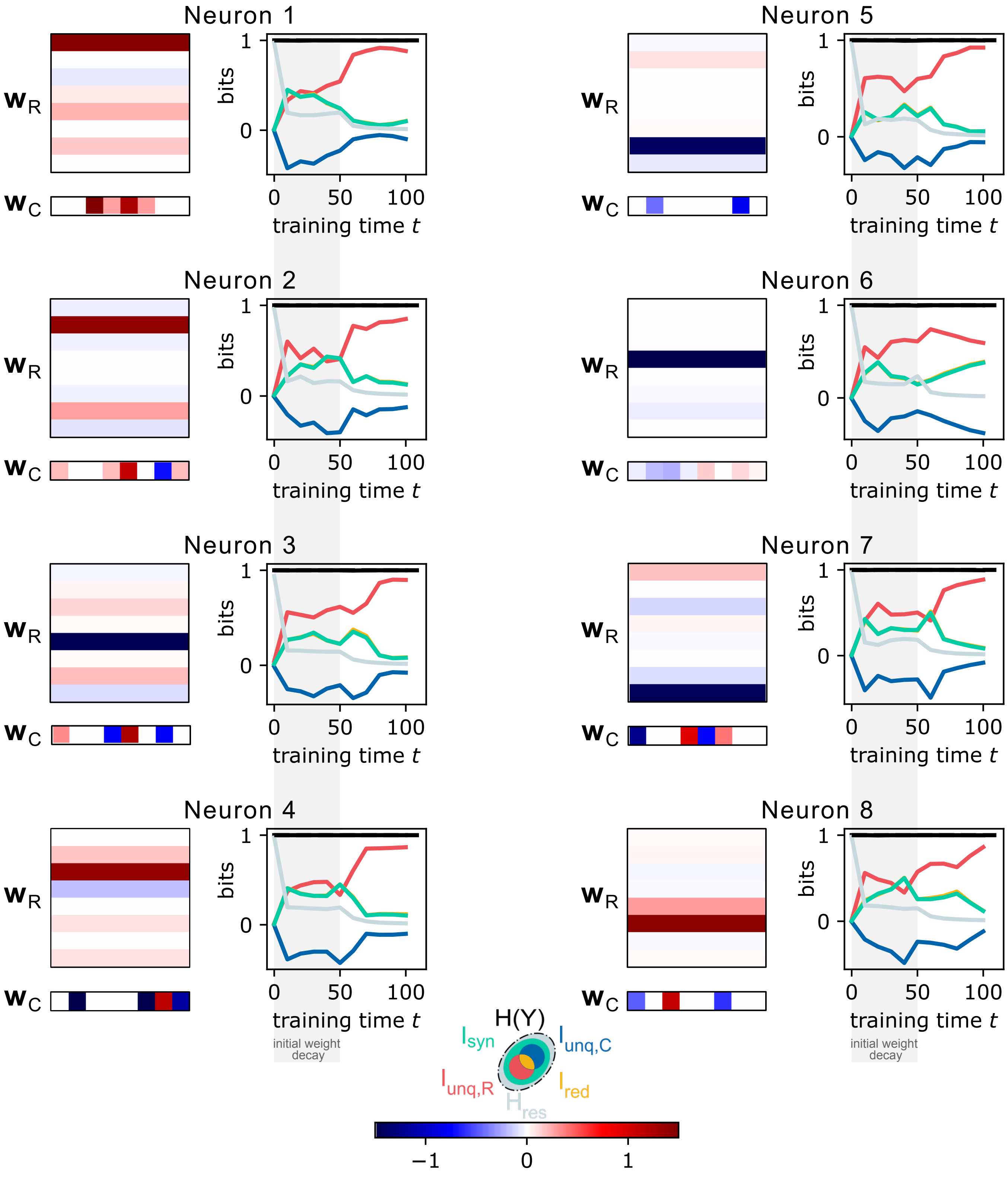

Fig. S4. Infomorphic neurons learn to encode distinct input features by unsupervised maximization of $I_{\mathrm{unq},R}$. The receptive fields $\mathbf{w}_R$ and contextual fields $\mathbf{w}_C$ (first and third columns), and the evolution of all information contributions over learning (second and fourth columns) are shown for each neuron of a randomly chosen network *successfully* performing unsupervised learning. The receptive input consists of 8 horizontal bars in an 8-by-8 grid, each bar appearing with probability $p = 0.5$. The contextual input is a vector of length 7, transmitting the output from all other neurons, without self-connections. Note that the values of all receptive and contextual weights are on the same scale indicated at the bottom of the figure.

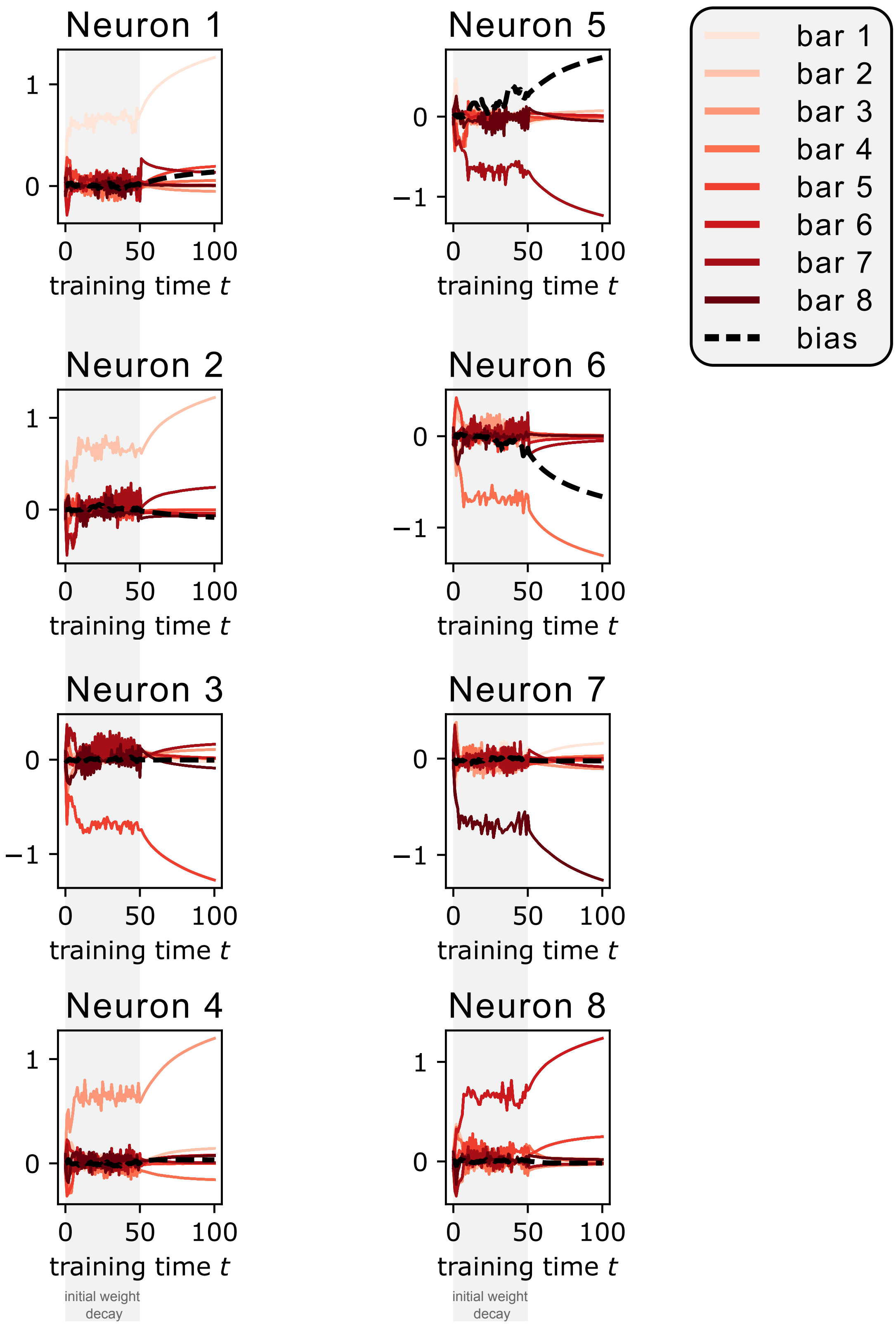

Fig. S5. Weight decay induces competition between neurons for individual bars. The temporal evolution of the receptive weights is shown for each neuron of the same network as in Figure S4 (dashed black line is the bias). Each neuron receives 8 receptive inputs from each bar (one per pixel), yet as the pixel values are perfectly correlated, the 8 weights per neuron and bar follow the same gradient and almost perfectly overlap in the figure, where we plot them in the same color.

All other network sizes show high redundant information I_{red}, despite the network with 60 patterns exhibiting low readout accuracy, taken as a sign of being above capacity. The high redundant information even above the capacity limit is compensated

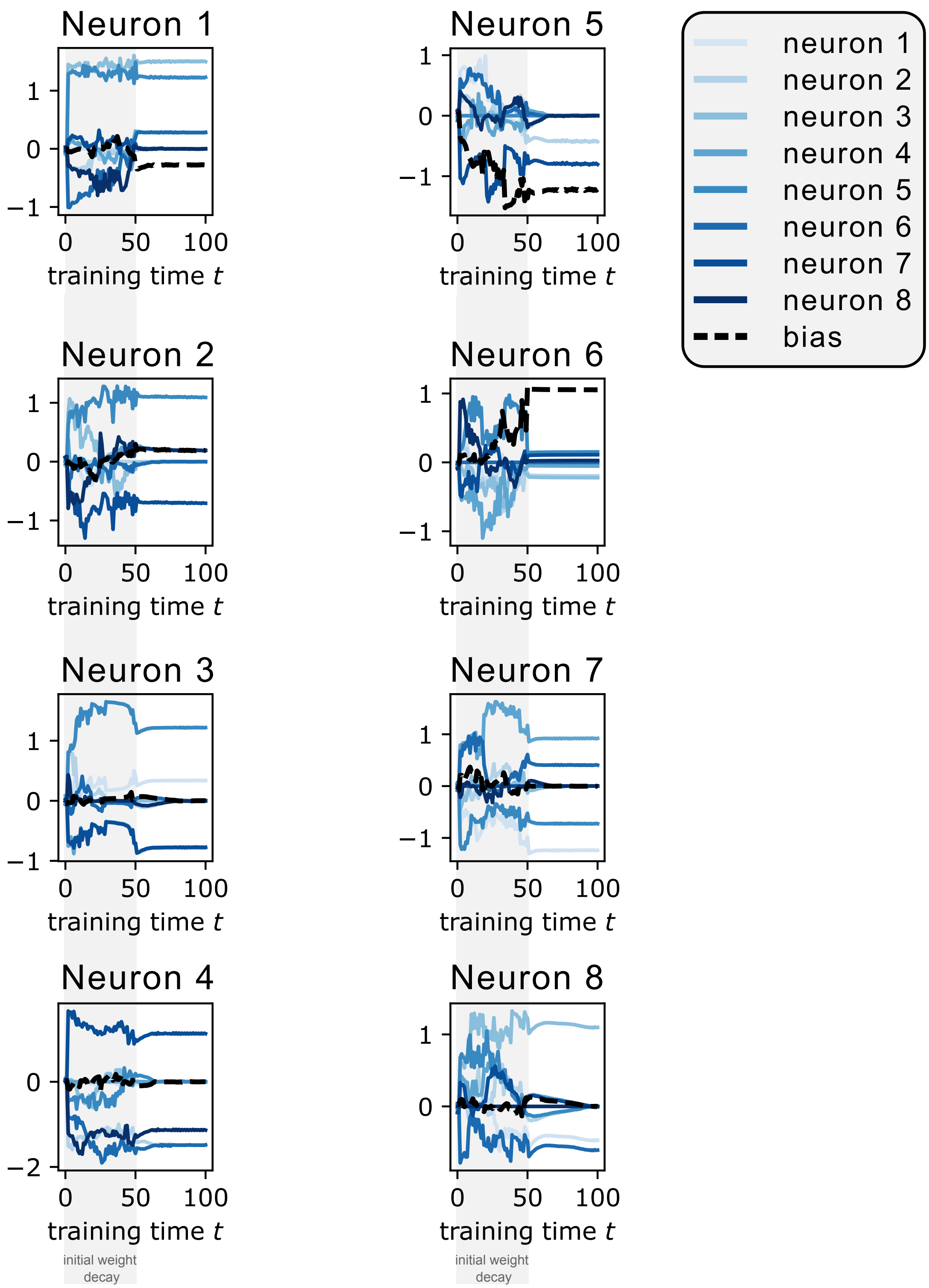

Fig. S6. Contextual weights almost exclusively change in the first phase of learning. The temporal evolution of the contextual weights is shown for each neuron of the same network as in Figure S4 (dashed black line is the bias). There are no contextual self-connections in the network.

by unique contextual misinformation, i.e. $I_{\mathrm{unq},C} < 0$. Negative information terms are possible in the $I_\cap^{\mathrm{sx}}$ measure, however, the consistency equation $I(Y:C) = I_{\mathrm{red}} + I_{\mathrm{unq},C}$ must still be satisfied. This results in low mutual information $I(Y:C)$ despite high redundant information I_{red}, indicating that the recurrent weights are in general not sufficient to predict the activity of the neuron in these networks, and thus firing patterns cannot be reliably sustained without external input.

Given that infomorphic networks exhibit a higher capacity than classical Hopfield networks trained with one-shot outer-product Hebbian learning (as shown in the main text), we calculate two further measures to compare these learning rules.

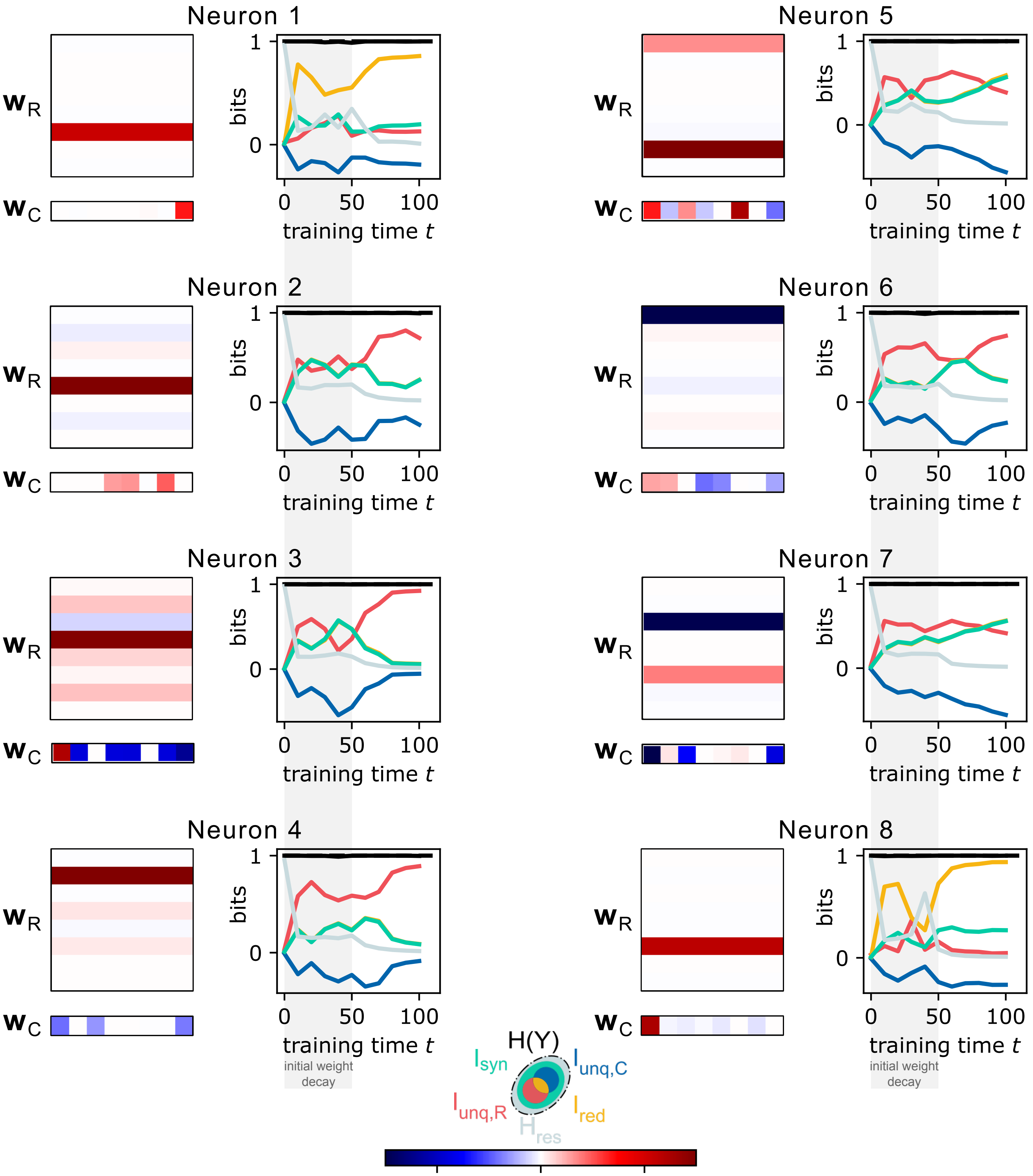

Fig. S7. Rare errors of two neurons encoding the same bar result in high redundant information I_{red}**.** The receptive fields $\mathbf{w}_R$ and contextual fields $\mathbf{w}_C$ (first and third columns), and the evolution of all information contributions over learning (second and fourth columns) are shown for each neuron of a randomly chosen network *unsuccessfully* performing unsupervised learning. Note that this is one of the two networks that encoded only 7 out of the 8 available bars. Additionally note that all the receptive and contextual weight fields are on the same scale indicated at the bottom of the figure.

Firstly, we calculate the cosine similarity between the weight matrix obtained from infomorphic networks and the weight matrix obtained from outer-product Hebbian learning on the same memory patterns. Given that outer-product Hebbian learning leads

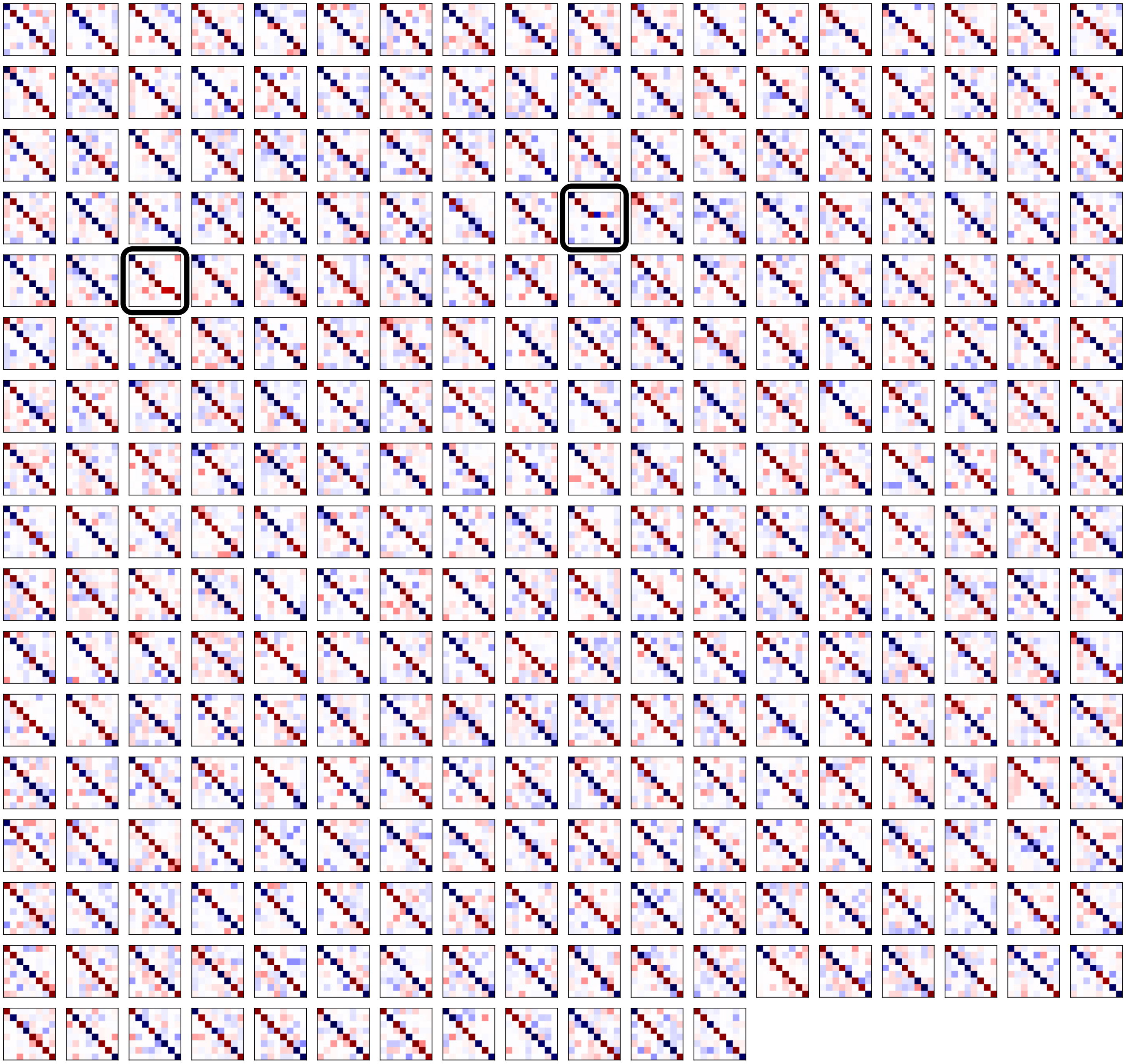

Fig. S8. Two out of 300 unsupervised infomorphic networks failed to encode all eight bars. We show a compressed version of the receptive fields for all 300 networks. All eight receptive weights connecting the pixels of each bar to each neuron highly overlap, so we only report the weight of the left-most pixel from each bar to each neuron. This results in an 8-by-8 matrix summarizing the receptive fields of all neurons in a network (rows are bars, columns are neurons). We sort the neurons by their preferred bars, such that dark pixels along the diagonal indicate perfect encoding. The two networks with sub-optimal encoding are framed in black.

to a symmetric weight matrix, we secondly calculate the symmetry of the obtained infomorphic weight matrix, as the cosine distance between the weight matrix and its transpose. We find consistent deviations both from perfect similarity and from perfect symmetry, which seem to get smaller for larger training sets (Figure S9). Further investigation of this phenomenon and comparisons to previously published online learning rules for associative learning are left for future work.

Finally, readout accuracy increases over training time, and networks with 12 and 35 patterns show almost instantaneous convergence to the correct memory pattern over readout time (Figure S9). In contrast, networks with 4 patterns show stable readout accuracy below 1, hinting at a stable sub-network that reaches a fixed point, in line with the idea that some neurons cannot learn due to lack of information in their receptive inputs. On the contrary, the readout accuracy for networks with 60 patterns decreases gradually over readout time, indicating an inability to sustain the cued pattern. Note that we do not binarize our readouts but keep the stochastic sampling, which leads to an unfair comparison, even impeding performance of the infomorphic networks in comparison to cla ssical Hopfield networks.

4. Supplementary Experiments

A. Primitive error neurons. In this section we construct simple "error neurons", as might later be used in infomorphic approaches to predictive coding (6, 7).

Topology and Inputs We train 25 infomorphic neurons with correlated scalar binary inputs as in the previous section, i.e., the receptive and contextual inputs to the neuron are jointly distributed as a two-dimensional Bernoulli distribution $p(X_R, X_C)$ with $p(X_R = -1, X_C = -1) = p(X_R = 1, X_C = 1) = .4$ and $p(X_R = -1, X_C = -1) = p(X_R = 1, X_C = 1) = .1$, resulting in a Pearson correlation of .6. As in the supervised learning experiments from the main text, the neurons are not connected to each other, as that would require a third input that is currently lacking.

Goal Functions We set the goal function of all neurons to $G = I_{\text{syn}} + I_{\text{unq},R} + I_{\text{unq},C} - I_{\text{red}}$. Intuitively, a perfect model would have maximal mutual information with the observations, such that an error neuron could then only extract redundant information between prediction and observation. As a perfect model should not be changed, in this case an optimal error neuron should have zero entropy. To achieve that, error neurons need to explicitly minimize redundant information I_{red}. All other information atoms correspond to a mismatch between model and observation, so should be maximized. Specifically, maximizing conditional information of the receptive input $I(Y : R \mid C) = I_{\text{syn}} + I_{\text{unq},R}$ makes the neuron encode for all information that is observed but has not been predicted. Simultaneously maximizing contextual unique information $I_{\text{unq},C}$ makes the neuron encode all information that has been predicted but not observed, thus providing a pruning signal to the model to not make unnecessary predictions.

Activation Functions We choose a simple symmetric activation function $A(r, c) = r + c$ because errors neurons should be driven by either input. Also this allows us to reduce each neuron to three trainable parameters w_R, w_C and bias $= w_{R,0} + w_{C,0}$, such that the full learning trajectories can be graphically depicted.

Protocol We train each neuron on 200 batches of 1000 randomly sampled stimuli. After each batch, we update the parameters (See Table S4 for all chosen training parameters).

Performance and Outcome Neurons learn to code for one of the infrequent events in the distribution, i.e. either $X_R = -1, X_C = 1$ or $X_R = 1, X_C = -1$, at random. This is intuitively correct, as these infrequent events signal sub-optimal prediction: An optimal binary prediction would perfectly correlate to the binary observation, such that $p(X_R = -1, X_C = 1) = p(X_R = 1, X_C = -1) = 0$. Note that an infomorphic neuron with a linear decision boundary cannot encode both infrequent events. However, a downstream prediction neuron might listen to multiple binary error neurons to get a full signed prediction error. We speculate that a ternary neuron with an output alphabet of three elements could be trained in a similar manner to code for both infrequent events. Alternatively, infomorphic neurons with three input classes could coordinate laterally, as in the unsupervised learning experiments. We furthermore speculate that such three-input-classes infomorphic neurons could also be used to signal prediction errors when the observations and model predictions are more high-dimensional and errors can exceed one bit of information.

Information Dynamics As expected, synergistic and unique information atoms increase over learning, and redundant information decreases. Furthermore, the neurons become less stochastic, as seen by a decrease of residual entropy. Importantly, the entropy $H(Y)$ of the error neurons decreases over learning, indicating that the amount of encodable "error information" is smaller than one bit, and that the neurons are indeed not encoding any redundant information or noise.

References

1. AJ Gutknecht, M Wibral, A Makkeh, Bits and pieces: Understanding information decomposition from part-whole relationships and formal logic. *Proc. Royal Soc. A* **477**, 20210110 (2021).
2. A Makkeh, AJ Gutknecht, M Wibral, Introducing a differentiable measure of pointwise shared information. *Phys. Rev. E* **103**, 032149 (2021).
3. J Kay, Information-theoretic neural networks for unsupervised learning: mathematical and statistical considerations, (Scottish Agricultural Statistics Service), Technical Report 1573387449478062080 (1994).
4. A Mohapatra, Logistic regression from scratch: Multi classification with onevsall (Analytics Vidhya, Medium) (2020).

Table S4. ***Parameters of the infomorphic neurons in the Primitive error neurons experiment.***

Training		
Parameter	Value	Comment
Phases	200	a single phase of training with 200 epochs
N_{tr}	1000	sampled from the set of $x, z \in \{-1, 1\}$
N_{te}	10000	sampled from the set of $x, z \in \{-1, 1\}$
m_{rep}	1	no repetition is needed
**Learning**		
Parameter	Value	Comment
b_{init}	0.5	all weights and biases initialized in $[-0.5, -0.125] \cup [0.125, 0.5]$, no qualitative difference for initialization in $[-0.5, 0.5]$
$\mathbf{\Gamma}$	(1, 1, -1, 1, 0)	goal parameters ($\Gamma_{\mathrm{unq},R}$, $\Gamma_{\mathrm{unq},C}$, Γ_{red}, Γ_{syn}, Γ_{res})
η	1	learning rate
λ	0.0	no weight pullback
**Input Integration**		
Parameter	Value	Comment
$n_{\mathrm{receptive}}$	1	$x \in \{1, -1\}$
$n_{\mathrm{contextual}}$	1	$z \in \{1, -1\}$
J_R	$[-8, 8]$	different range does not affect the training
J_C	$[-8, 8]$	different range does not affect the training
n_R-bins	500	uniform bin-size is 0.032
n_C-bins	500	uniform bin-size is 0.032

5. JJ Hopfield, Neural networks and physical systems with emergent collective computational abilities. *Proc. national academy sciences* **79**, 2554–2558 (1982).
6. RP Rao, DH Ballard, Predictive coding in the visual cortex: a functional interpretation of some extra-classical receptive-field effects. *Nat. neuroscience* **2**, 79–87 (1999).
7. B Millidge, A Tschantz, CL Buckley, Predictive coding approximates backprop along arbitrary computation graphs. *Neural Comput.* **34**, 1329–1368 (2022).

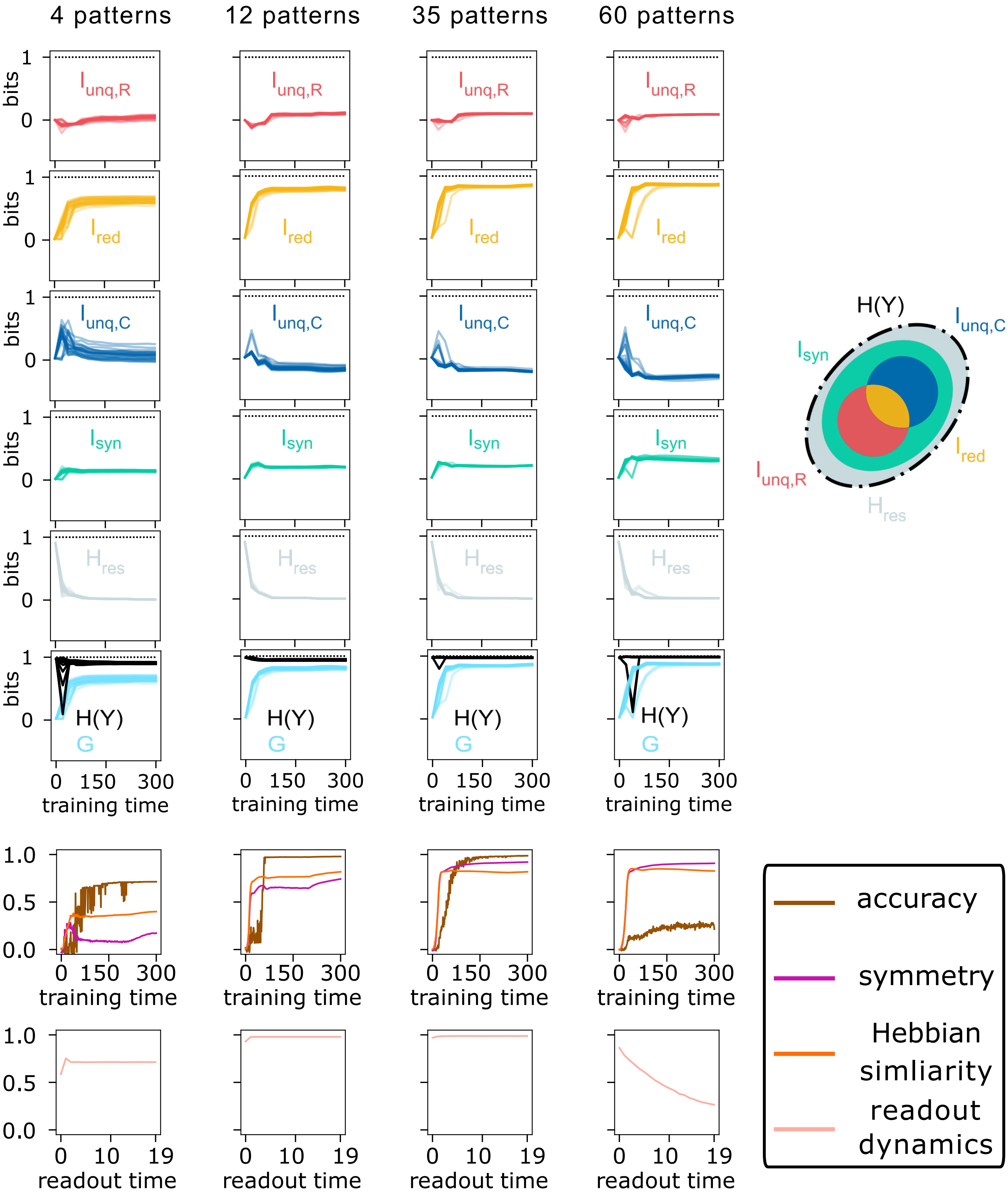

Fig. S9. Maximizing I_{red} leads to an asymmetric learning rule for auto-associative memories, which is different from outer-product Hebbian learning. In the first six rows, we show the evolution of the PID information atoms $I_{\mathrm{unq},R}$, I_{red}, $I_{\mathrm{unq},C}$ and I_{syn}, the residual entropy $H(Y \mid R, C)$, the full output entropy $H(Y)$ and the goal function $G = 0.1 I_{\mathrm{unq},R} + 0.1 I_{\mathrm{unq},C} + I_{\mathrm{red}} + 0.1 I_{\mathrm{syn}}$ over time. Each information quantity is averaged over all 100 neurons in a network, for each of 25 networks trained on either 4, 12, 35, and 60 patterns (columns). In the fifth row, we show for one randomly chosen networks of each size: the cosine similarity of the readout pattern with the correct memory pattern after 20 time steps ("accuracy"), the cosine similarity of the recurrent contextual weight matrix W_C and its transpose ("symmetry"), and the cosine similarity of W_C with the corresponding outer-product Hebbian weight matrix (5) ("Hebbian similarity") over training time. In the sixth row, we plot for the same networks the cosine similarity of the readout pattern with the correct memory pattern over the 20 time steps of the readout. The cue pattern is presented only in the first time step, afterwards the receptive input is set to $\mathbf{x}_R = 0$.

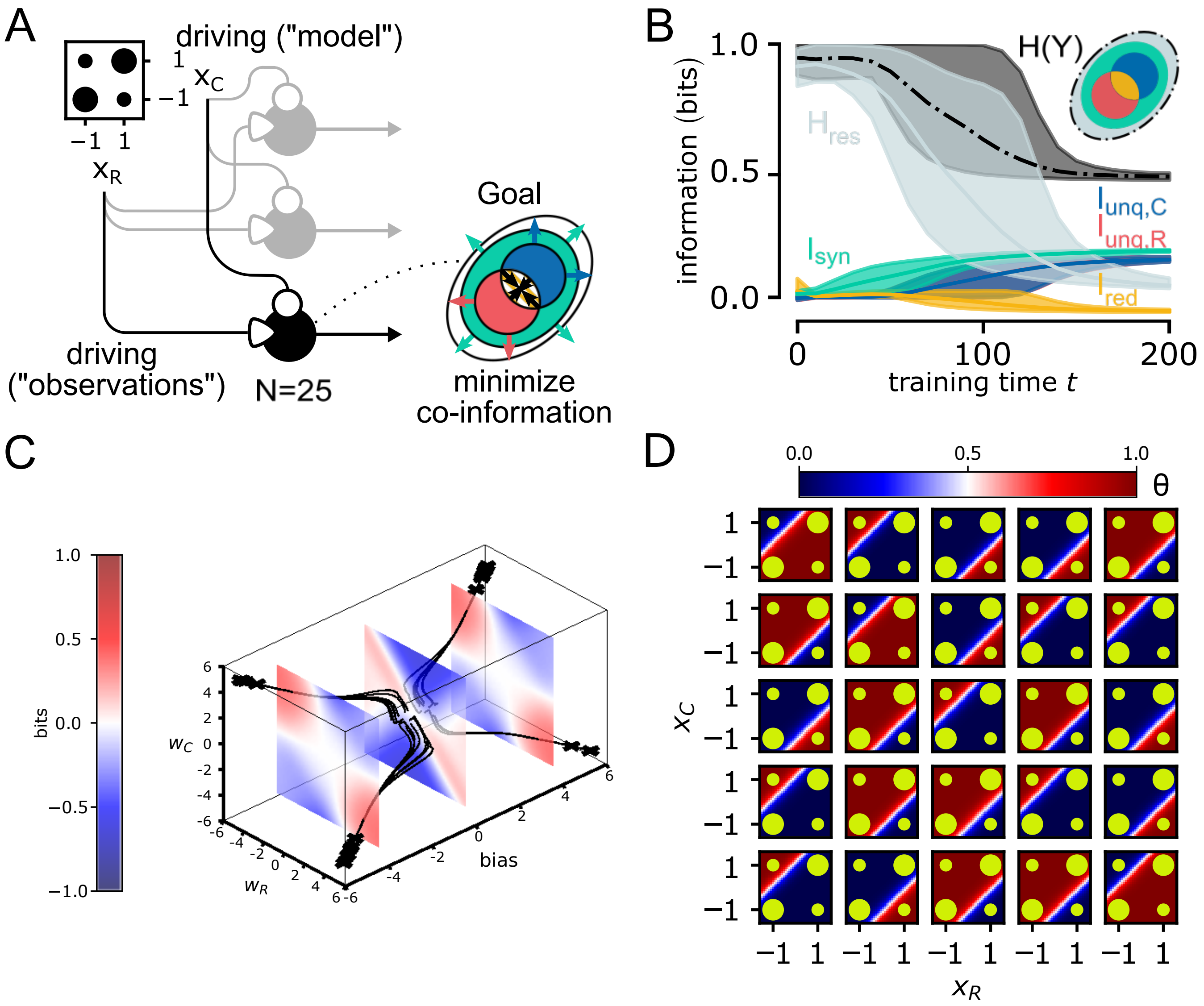

Fig. S10. Binary infomorphic error neurons via maximization of synergy and uniqueness and minimization of redundancy. By maximizing $G = I_{\mathrm{syn}} + I_{\mathrm{unq},R} + I_{\mathrm{unq},C} - I_{\mathrm{red}}$ between two binary, correlated inputs, infomorphic neurons learn to code for rare events, resulting in signed error neurons. (A) Network architecture for a set of neurons that do not communicate laterally, each receiving the same scalar binary inputs $\mathbf{X}_R$ (the "observations") and $\mathbf{X}_C$ (the "model"). The inputs are both Bernoulli-distributed with $p = p(X_R = 1) = p(X_C = 1) = .5$, and correlated with $p(X_R = X_C = 1) = p(X_R = X_C = -1) = .4$ and $p(X_R = -X_C = 1) = p(-X_R = X_C = 1) = .1$. Choosing the symmetric activation function $A(r, c) := r + c$ gives equal importance to model and observations, and results in a single bias term of the form $\mathrm{bias} = w_{0,R} + w_{0,C}$. (B) Information quantities and goal function averaged over all 25 independently trained neurons. Shaded areas correspond to the 95-percentile. (C) Graphical depiction of the full learning dynamics. Black lines show trajectories in parameter space over the course of learning, with the endpoint of learning indicated by a "+" marker. Depending on their random initialization, neurons pick one of four corners of the three-dimensional parameter cuboid. Colored surfaces show the goal function G for different values of the weights at fixed bias. (D) Boundaries in input space for all 25 neurons after training. Background color indicates firing probability $\theta = p(Y = 1 \mid X_R, X_C)$, green disks denote the input distribution, with the area of a disk corresponding to the probability of the respective event (compare with panel A). The neurons randomly choose one of the two rare events and learn to separate it from all other events. Taking multiple error neurons into account, a downstream neuron could use this as a signed error indicator detecting disagreement between the model and the observations.
